# The two-dose MVA-BN mpox vaccine induces a nondurable and low avidity MPXV-specific antibody response

**DOI:** 10.1128/jvi.00253-25

**Published:** 2025-03-31

**Authors:** Aaron L. Oom, Kesi K. Wilson, Miilani Yonatan, Stephanie Rettig, Heekoung Allison Youn, Michael Tuen, Yusra Shah, Ashley L. DuMont, Hayley M. Belli, Jane R. Zucker, Jennifer B. Rosen, Ramin Sedaghat Herati, Marie I. Samanovic, Ralf Duerr, Angelica C. Kottkamp, Mark J. Mulligan, on behalf of the NYC OSMI Study Group

**Affiliations:** 1 New York University Langone Vaccine Center, Department of Medicine, New York University Grossman School of Medicine 214143, New York, New York, USA; 2 Department of Host-Microbe Interactions, St. Jude Children’s Research Hospital 5417, Memphis, Tennessee, USA; 3 Bureau of Immunization, New York City Department of Health and Mental Hygiene, Queens, New York, USA; Northwestern University Feinberg School of Medicine, Chicago, Illinois, USA

**Keywords:** vaccination, MVA-BN, mpox, MPXV, antibody response, JYNNEOS, orthopoxviruses

## Abstract

The 2022 global outbreak of clade IIb mpox was the first major outbreak of mpox outside of African nations. To control the outbreak, public health officials began vaccination campaigns using the third-generation orthopoxvirus vaccine modified vaccinia Ankara from Bavarian Nordic (MVA-BN). Prior to this outbreak, the durability of monkeypox virus (MPXV)-specific immunity induced by MVA-BN was poorly understood. In 2022, we launched the New York City Observational Study of Mpox Immunity (NYC OSMI, NCT05654883), a longitudinal study of 171 participants comprising MVA-BN vaccines and mpox convalescent individuals. Peripheral blood sampling was performed at intervals including prior to vaccination, after one dose, and after the second dose. MVA-BN vaccinees with and without a history of smallpox vaccination demonstrated detectable MPXV-specific memory B cells at 1-year post-vaccination. Additionally, MVA-BN increased MPXV neutralizing titers in smallpox vaccine-naïve vaccinees, with a comparable maximum titer reached in naïve and smallpox vaccine-experienced vaccinees. However, neutralizing titers returned to baseline within 5–7 months for naïve individuals, while remaining elevated in those with prior smallpox vaccination. Both naïve and experienced individuals generated robust IgG responses against MPXV H3 and A35, but naïve vaccinees’ IgG responses showed lower avidity than experienced vaccinees. These data highlight a low avidity antibody response elicited by MVA-BN that is short-lived in naïve vaccinees. This work supports the need for long-term studies on protection induced by MVA-BN, including the potential need for booster doses as well as the development of next-generation orthopoxvirus vaccines.

**IMPORTANCE:**

The ongoing outbreaks of mpox demonstrate the continuing threat of orthopoxviruses to global health. While previous orthopoxvirus vaccines generated lifelong antibody and cellular immunity, we show here that the current mpox vaccine, MVA-BN or JYNNEOS, fails to induce durable antibody immunity in individuals with no prior smallpox vaccination. This raises the important question of whether MVA-BN vaccinees have long-term protection from mpox. Our work highlights the need for further studies into the durability of protection generated by MVA-BN as well as whether subsequent booster doses are necessary to maintain protection.

## INTRODUCTION

The 2022 global outbreak of clade IIb mpox (formerly known as monkeypox) was the first major outbreak of mpox outside of African nations, with nearly 100,000 cases reported to date primarily in Europe and the Americas (1). Over 34,000 cases have occurred in the US alone (2). With cases primarily clustered among men who have sex with men, it was clear that sexual transmission was playing a larger role in this outbreak (3) compared to transmission from zoonoses or direct contact typically noted in endemic cases (4). To stem rising case numbers worldwide, public health agencies launched awareness campaigns and began offering the two-dose MVA-BN vaccine series (Bavarian Nordic). MVA-BN (also known as IMVAMUNE, IMVANEX, or JYNNEOS) is an FDA-approved modified vaccinia Ankara vaccine indicated for the prevention of mpox when administered twice subcutaneously (SC) over 28 days. MVA-BN is a third-generation nonreplicating orthopoxvirus vaccine, in contrast to replication-competent vaccinia virus (VACV)-based first- and second-generation vaccines (e.g., DryVax or ACAM2000, respectively).

At the time of the 2022 outbreak, several studies had clearly established the decades of robust immunity generated by VACV vaccination (5–9), but studies of MVA-BN durability had yielded more mixed results. Ilchman et al. found that VACV-specific neutralizing titers wane to baseline levels within 2 years (10), while Priyamvada et al. reported higher than baseline monkeypox virus (MPXV)- and VACV-specific neutralizing titers at 2 years post-vaccination (11). However, the latter study was conducted in the Democratic Republic of Congo where mpox is endemic, which may have impacted participant immunity. We still lack insight into key areas of MPXV-specific antibody biology, including antibody specificities, avidity, and durability. Subsequent studies of the antibody repertoire following MVA-BN vaccination showed that MPXV antigen-specific antibodies were maintained at 2–3 months, but whether the titers would persist long-term remains unknown (12, 13).

To begin addressing knowledge gaps within MPXV-specific immunity following MVA-BN vaccination, we designed and conducted the New York City Observational Study of Mpox Immunity (NYC OSMI or OSMI). OSMI is a longitudinal study of MPXV-specific immunity in MVA-BN vaccinees and individuals with prior MPXV infection in NYC that began specimen collection in July 2022. We previously reported an interim analysis of OSMI where we observed a marked difference in binding antibody responses between MVA-BN vaccinees that had no prior smallpox vaccination (naïve vaccinees) and those that did (experienced vaccinees) (14). Since then, there have been additional reports from other groups that further highlighted the differences between these two groups as well as the limited durability of MPXV- or VACV-specific binding and neutralizing antibodies in naïve MVA-BN vaccinees (15–18).

In further characterization of these humoral responses, we observed that following vaccination, the binding antibody titers that correlated with MPXV neutralization were low avidity and showed little to no avidity maturation by 1-year post-dose two in naïve vaccinees. However, both naïve and experienced vaccinees demonstrated detectable levels of MPXV-specific memory B cells, which may prove protective against subsequent exposures. These data highlight a nondurable, low avidity response from the two-dose regimen of MVA-BN in naïve people that warrants further study of booster doses (10, 19) and correlates of protection, more broadly.

## MATERIALS AND METHODS

### Study design

NYC OSMI investigates the MPXV-specific immune response in adults with and without HIV who have received MVA-BN vaccination as well as those who have recovered from mpox disease (NCT05654883, Institutional Review Board protocol 22-01338). Participants underwent sampling for serum, peripheral blood mononuclear cells (PBMCs), and saliva at specified intervals: baseline, ~30 days post-dose one, and ~3 weeks, ~3 months, and ~9-10 months post-dose two. Participants enrolled either pre- or post-MVA-BN vaccination, with the enrollment window extending up to 365 days post-first dose or mpox symptom onset. One hundred seventy-one adult participants consented to be evaluated. Participants were recruited from city vaccination centers as well as by word of mouth. Cohort demographics and clinical information for participants analyzed here can be found in Table 1; additional details on mpox convalescent subjects are in Table S1.

**TABLE 1:** Demographics of the NYC Observational Study of Mpox Immunity

	Total MVA-BN-vaccinated participants		Prior smallpox vaccination (experienced)		No prior smallpox vaccination (naïve)	
	**No**.	**%**	**No**.	**%**	**No**.	**%**
Enrolled participants	159		49	30.8	110	69.2
Gender						
Male	128	80.5	46	93.9	82	74.5
Female	18	11.3	3	6.1	15	13.6
Nonbinary/gender fluid/queer	13	8.2	0	0.0	13	11.8
Age						
Median	38		62		33	
Range			46 to 75		20 to 50	
Race/ethnicity						
White	104	65.4	39	79.6	65	59.1
Black or African American	15	9.4	6	12.2	9	8.2
Asian	17	10.7	2	4.1	15	13.6
Other	23	14.5	2	4.1	21	19.1
Non-Hispanic	118	74.2	40	81.6	78	70.9
Hispanic	39	24.5	8	16.3	31	28.2
Other	2	1.3	1	2.0	1	0.9
LGBTQ+						
Yes	142	89.3	46	93.9	96	87.3
No	17	10.7	3	6.1	14	12.7
Administration route						
SC-SC	35	22.0	10	20.4	25	22.7
ID-ID	31	19.5	13	26.5	18	16.4
ID-SC	16	10.1	8	16.3	8	7.3
SC-ID	66	41.5	16	32.7	50	45.5
Incomplete/unknown	11	6.9	2	4.1	9	8.2
HIV status						
PWH	42	26.4	26	53.1	16	14.5
HIV-uninfected	117	73.6	23	46.9	94	85.5

Prior smallpox vaccination status was assessed through an adjudication process. For this process, the following factors were considered: physical examination of the upper arms for the presence of scar consistent with past vaccinia vaccination, prior Bacillus Calmette-Guerin vaccination, prior military service or other occupational risk requiring smallpox vaccination (including research laboratory work), country of birth, year of migration to the US, and whether a participant’s year of birth was consistent with active smallpox vaccine distribution in the participant’s country of origin. A literature review was performed to determine when individual countries ended their routine smallpox vaccination campaigns, e.g., in 1972 in the US. Reviews of smallpox vaccination status were conducted by two infectious disease physicians.

For people with HIV (PWH), CD4+ T cell counts were obtained from electronic medical records at the time of enrollment (Table S2). The date and route of mpox vaccination were verified through electronic medical records, participant’s description of the vaccination procedure, and/or records available through the New York City Health Department’s Citywide Immunization Registry.

Additional pre-2022 control samples and pre-OSMI enrollment participant samples were obtained from the NYU Langone Vaccine Center Biorepository (Institutional Review Board protocol 18-02035).

### Blood collection

Venous blood was collected by standard phlebotomy. PBMCs were isolated from CPT vacutainers (BD Biosciences) and processed within 4 h of collection. PBMCs were viably cryopreserved and thawed later for assays. Sera were collected in SST tubes (BD Biosciences), aliquoted, and frozen immediately at −80°C.

### MPXV virus stock propagation

All MPXV work was conducted in a certified ABSL3 facility at NYU Grossman School of Medicine. MPXV clade IIb, lineage B.1, was obtained from BEI Resources (NR-58622); this virus was isolated in Massachusetts, USA during the 2022 outbreak. Working stocks of MPXV were generated by plaque purification in Vero E6 cells (ATCC #CRL-1586) followed by three passages of propagation in Vero E6 cells at a multiplicity of infection (MOI) of 0.01 for 3 days each at 37°C. Virus was harvested by freeze-thaw lysis of scraped cells followed by centrifugation at 1,000 × *g* to remove cell debris. At each passage, stocks were purified over 36% sucrose in TNE buffer (Quality Biological #351-302-101) at 32,900 *× g* for 80 min at 4°C. Purified MPXV stocks were sequence-verified.

### Immunofluorescence-based MPXV microneutralization assay

One day before MPXV infection, 1.5 × 10^4^ Vero E6 cells were plated in each well of a black 96-well assay plate in cell culture media (Dulbecco’s Modified Eagle Medium [DMEM] supplemented with penicillin/streptomycin, 2 mM L-glutamine, and 10% heat-inactivated fetal bovine serum [FBS]) and incubated at 37°C and 5% CO_2_. All participant sera were complement-inactivated prior to use by heating for 30 min at 56°C. Participant serum was twofold serially diluted in infection media (DMEM lacking sodium pyruvate supplemented with 2% heat-inactivated FBS) starting at a 2.5-fold dilution (final starting dilution is fivefold following the addition of an equal volume of virus) for 8-points; all dilutions were performed in triplicate. Prior to infection, serum dilutions were incubated with MPXV (sufficient for MOI = 0.01) for 1 h at 37°C. Cells were washed with 1× phosphate-buffered saline (PBS) and then incubated with serum-virus mixtures for 42 h at 37°C and 5% CO_2_. Following infection, plates were submerged in 10% formalin (Fisher Scientific #SF984) for 1 h at room temperature (RT). Fixed samples were rinsed with water then permeabilized and blocked with 3% bovine serum albumin (BSA) in PBS (blocking buffer) with 0.1% Triton X-100 for 30 min at RT. Samples were then incubated with a polyclonal rabbit anti-vaccinia virus Lister strain antibody (Abbexa #abx023200) diluted 1:1,000 in blocking buffer for 1 h at RT. All plates were washed four times with PBS and then stained with 1:2,000 dilution of donkey anti-rabbit AlexaFluor647 secondary (Thermo #A-31573) and 1.25 µg/mL DAPI in blocking buffer. Following staining, cells were washed four times with 1× PBS before filling each well with 100 µL of PBS for imaging and quantification using the BioTek Cytation 7 Cell Imaging Multi-Mode Reader and Gen5 Image Prime software. MPXV neutralizing titers (ID_50_ values) were calculated from percent inhibition using GraphPad Prism 10.0.03 nonlinear regression (variable slope with four parameters) with top and bottom constraints (100 and 0, respectively). Samples with ID_50_ values beyond the initial dilution range were repeated with an adjusted dilution curve.

### Multiplexed immunoassay for binding antibodies and avidity

Binding and avidity of MPXV-specific IgG in serum were measured using the Luminex platform for a 12-plex assay. Eight different MPXV proteins were included: A29 (Sino Biological #40891-V08E), A30 (Cell Sciences #YVV15001A), A35 (Sino Biological #40886-V08H), B16 (Cell Sciences #YVV17401A), B21 (Cell Sciences #YVV16301A), E8 (Cell Sciences #YVV13201A), H3 (Sino Biological #40893-V08H1), and L1 (Sino Biological #40889-V07E). In addition, quality control beads were included: rubella virus (RUBV) E1/E2 (ACROBiosystems #GL2-R5583), BSA (Thermo Scientific #J65097.22), human recombinant IgG1 (Sigma-Aldrich #I5154), and IC45 (Luminex #MRP1-045-01). Differing bead regions were coupled to antigen using the xMAP Antibody Coupling Kit (Luminex #40-50016) at the following optimized concentrations: 10 pmol/1 × 10^6^ beads for H3, A35, RUBV E1/E2, and human IgG1; 50 pmol/1 × 10^6^ beads for E8 and A29; and 100 pmol/1 × 10^6^ beads for A30, B16, B21, L1, and BSA. All MPXV antigens were His-tagged, and coupling was confirmed using an anti-His antibody (Abcam #ab27025). Beads were multiplexed with 1,000 beads per region for each sample.

Samples were analyzed by first incubating equal volumes of complement-inactivated serum and multiplexed beads for 1 h in the dark on a shaker at RT. All sera were analyzed at final dilutions of 1:100 and 1:2,000, prepared in technical duplicate in 1× PBS (−Ca^2+^ −Mg^2+^) with 0.01% BSA and 0.02% Tween-20 (PBS-TB). The beads were then washed twice with PBS-TB before being incubated with either PBS (−Ca^2+^ −Mg^2+^) or the chaotropic agent, 2 M ammonium thiocyanate (NH_4_SCN) for 30 min in the dark on a shaker at RT; one technical replicate of each dilution was used for each condition. Beads were washed again and then incubated for 30 min as before with a biotinylated rabbit anti-human IgG H&L detection antibody (Abcam #ab97158) diluted to 2 µg/mL in PBS-TB. Finally, the beads were washed before a final incubation as before with streptavidin-PE (BioLegend #405204) diluted to 4 µg/mL in PBS-TB for 30 min. The beads were washed a final time and then run on a Luminex 200 instrument with a sample volume set to 50 µL. Instrument settings were as follows: 50 bead minimum per region and DD gate set 6,000 to 17,000. Binding titers were calculated by measuring the area under the curve (AUC) of the median fluorescence intensity of the two dilutions that received PBS, not NH_4_SCN. To calculate avidity, the binding AUC from the NH_4_SCN condition was divided by the AUC from the PBS condition to determine a ratio that is reported as the avidity index. Samples were normalized by the inclusion of a positive control pool on each plate, generated from pre-2022 control sera from participants with prior smallpox vaccination; normalization was conducted on an antigen-by-antigen basis.

### Anti-MPXV H3 enzyme-linked immunosorbent assay

Binding antibodies against MPXV H3 protein were measured via direct enzyme-linked immunosorbent assay (ELISA) of participant serum as previously described (14). In brief, 96-well plates were coated overnight at 4°C with 0.5 µg/mL MPXV H3 protein (Sino Biological Inc., 40893-V08H1) diluted in PBS; coated plates were washed with PBS-T prior to blocking. Plates were blocked with PBS +0.05% Tween 20 (Thermo Fisher Scientific) (PBS-T) +5% nonfat milk (blocking buffer) for 1 h at RT. Heat-inactivated serum samples were serially diluted in blocking buffer and incubated in wells for 2 h at RT. Horseradish peroxidase-conjugated goat-anti-human IgG (Southern BioTech, 2040-05) was diluted in blocking buffer (1:2,000) and added to each well to incubate for 1 h at RT. Finally, plates were developed for 5 min with 3,3′,5,5′-tetramethylbenzidine peroxidase substrate (Thermo Fisher Scientific) followed by 1 N hydrochloric acid to halt the assay. Absorbance at 450 nm was measured on a Synergy 4 (BioTek) plate reader. A reaction positivity cutoff was determined by calculating two times the standard deviation plus the mean of 16 pre-2022 control sera from subjects with no prior history of smallpox vaccination. Endpoint titers were calculated by interpolating at which reciprocal dilution a sample’s curve crossed the cutoff using the nonlinear fit analysis in GraphPad Prism 9.5.1. Normalization for batch effect was addressed by the inclusion of a positive control pool generated from pre-2022 controls with prior smallpox vaccination. Sera with titers below the limit of detection (50) were scored as 25.

### Memory B cell ELISpots

Memory B cells were quantified as previously described (20) with application-specific changes elucidated here. Briefly, cryopreserved PBMCs were thawed and resuspended in 1 mL of warmed RPMI-1640 media supplemented with 10% heat-inactivated FBS and 2 mM L-glutamine (R10 media). Cells were counted and resuspended at 1 × 10^6^ cells/mL in stimulation media (R10 supplemented with 100 U/mL of DNase I, 1:1,000 dilution of B-poly-S [Cellular Technology Limited #CTL-HBPOLYS-200], and 1 µM β-mercaptoethanol). A total of 2 × 10^6^ cells were added across two wells of a 24-well plate and incubated for 5 days at 37°C and 5% CO_2_. At least 18 h prior to day 6, Millipore 96-well multiscreen HA filter plates (Millipore #MSHAN4B50) were coated with 2 µg/mL of recombinant MPXV H3 or A35, 10 µg/mL of donkey anti-human IgG Fcγ fragment (Jackson ImmunoResearch # 709-005-098), or 4.16 µg/mL of Imject Mariculture keyhole limpet hemocyanin (KLH) (Thermo Scientific #77600), then incubated at 4°C. On day 6, coated assay plates were washed four times with 1× PBS-T and then blocked with R10 media at 37°C and 5% CO_2_ for 1–2 h. During the blocking, stimulated PBMC cultures were transferred to 15 mL conical tubes, washed with warmed R10 media, then resuspended in R10 media at a concentration of 1 × 10^7^ cells/mL. A dilution series was prepared for each sample by adding 5 × 10^5^ cells to the first row of wells on the blocked assay plates, then serially diluting threefold for a 4-point dilution curve. For the total IgG plate, only 5 × 10^4^ cells were added. Plates were incubated for 6 h at 37°C and 5% CO_2_. Following incubation, plates were washed twice with PBS and then four times with PBS-T. Plates were then incubated overnight at 4°C with biotinylated donkey anti-human IgG Fcγ fragment (Jackson ImmunoResearch # 709-065-098) diluted 1:1,000 in PBS-T +2% FBS (antibody diluent). The next morning, plates were washed four times with PBS-T, then incubated with avidin-D-HRP (Vector Laboratories # A-2004-5) diluted 1:1,000 in antibody diluent for 1 h at RT. Plates were washed three times with PBS-T, then three times with PBS before developing for 5 min with AEC substrate (BD Biosciences #551951). Following the development, plates were washed twice with distilled water and then allowed to dry for ~24 h before imaging and spot counting on an ImmunoSpot automated ELISpot counter (ImmunoSpot v7.0.9.5, Cellular Technology Limited). Samples with less than ~750 IgG+ memory B cells per million PBMCs were removed from the analysis. Additionally, samples with a KLH signal more than three times the standard deviation over the mean KLH signal were removed as outliers. Spots were counted based on a size threshold determined by the counter software using KLH wells as negative controls and total IgG wells as positive controls. Only spots greater than 3,000 square microns were counted.

### Statistical methods

Paired measurements in figures were analyzed by the Wilcoxon matched-pairs signed rank test. Comparisons of multiple sample groups in figures were conducted by the Kruskal-Wallis test with Dunn’s method for multiple comparisons. Linear mixed-effects regressions (LMER) were used to model neutralizing titer durability with repeated measurements. The *lme4* package (v.1.1-35.1) in R v.4.3.2 was used for all LMER calculations. Models were fit using the natural log transformation of the neutralizing titers. Both participant-level random intercepts only and participant-level random intercepts and slopes models were tested, but random intercepts only models were ultimately used as random intercepts and slopes models either failed to converge or had insufficient data for model fitting. Model predictions were generated using the *ggeffects* package (v.1.3.2). Half-lives were calculated by rearrangement of the LMER to its exponential decay form and use of the predicted slope for the days post-dose two variables. LMER model parameters for each instance are detailed in the Supplementary Materials. Correlation testing, paired measurements, and multiple comparison testing were all conducted in GraphPad Prism v.10.0.3. For all figures, *P*-value <0.05 is *, <0.01 is **, and <0.001 is ***.

## RESULTS

### The New York City Observational Study of Mpox Immunity

**Fig 1:**
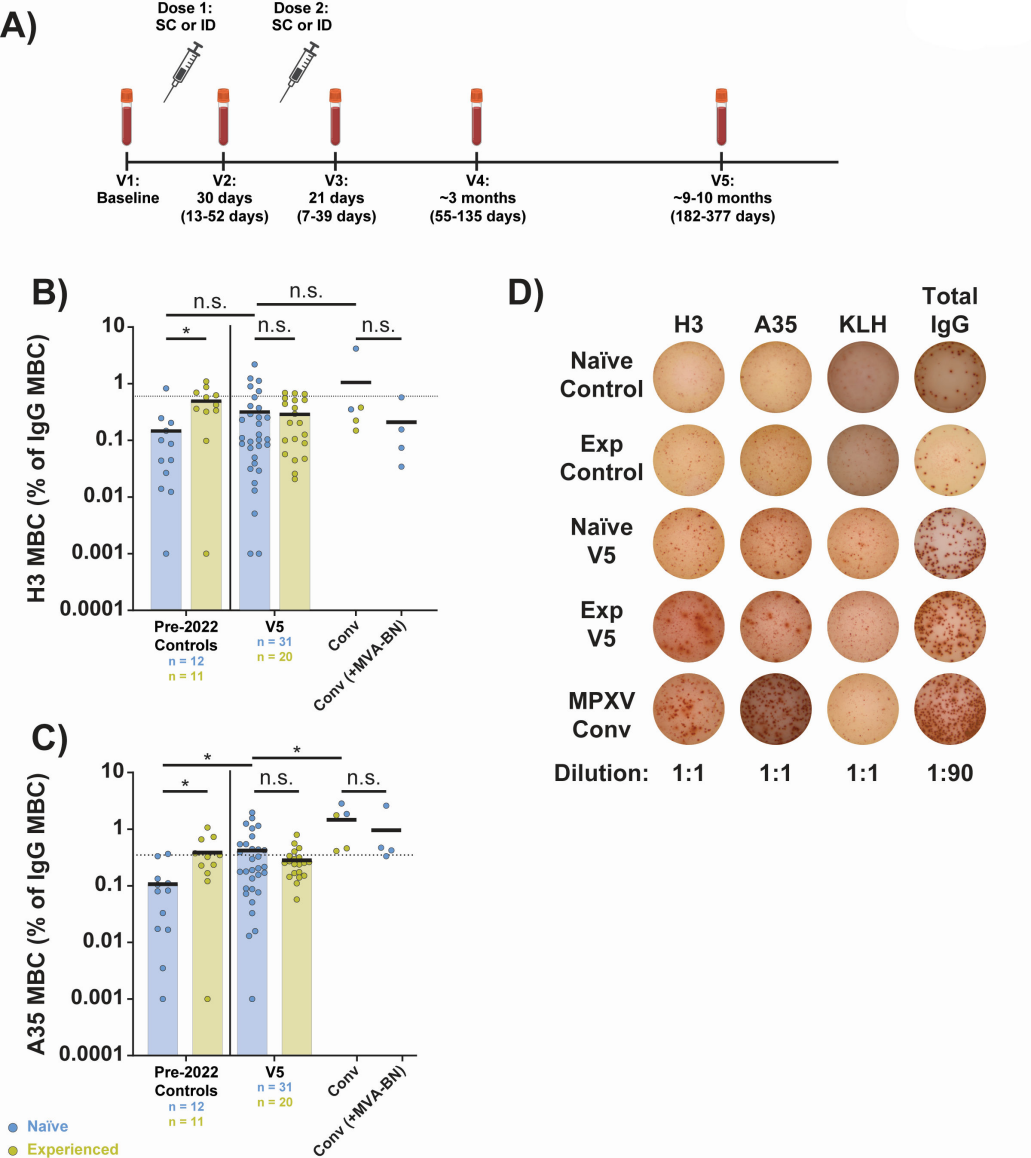
MPXV H3- and A35-specific memory B cells are detectable at 1 year after MVA-BN vaccination. IgG+ memory B cells (MBCs) were quantified via ELISpot. (**A**) Diagram of study visits. For each visit, the median days post-vaccination are shown along with the range of days used for each visit in parentheses. The windows of time denoted for each visit are used throughout the paper in analyses of specific visits. Diagram generated in BioRender. (**B**) Aggregate MPXV H3-specific MBCs as a proportion of total IgG+ MBCs. (**C**) Aggregate MPXV A35-specific MBCs as a proportion of total IgG+ MBCs. (**D**) Representative well images from ELISpot assay. Convalescent (conv) samples were taken at ~1 year post-mpox symptom onset. In panels B and C, colored/black bars indicate the mean. Black, dashed line in all panels is the positivity threshold for binding as determined by 12 pre-2022 negative controls (mean of controls plus two times the standard deviation). Statistical testing conducted by Kruskal-Wallis test with Dunn’s method for multiple comparisons for panels B and C. Samples with a value of 0 have been displayed as 0.001% to accommodate the log scale; statistics were calculated with actual value. SC, subcutaneous; ID, intradermal; Exp, experienced vaccinees (prior history of smallpox vaccination); KLH, keyhole limpet hemocyanin (negative control).

We report here on 159 MVA-BN vaccinees with or without a prior history of smallpox vaccination (Table 1) and nine mpox convalescent individuals (Table S1). The remaining three study participants were excluded due to missing data or visits. The study visit schedule is described in Fig. 1A; participants could enroll at any time point. Possible time points included a baseline visit (V1), a visit between vaccine doses (V2), and three visits following receipt of the second vaccine dose: median visit dates of ~3 weeks post-dose two (V3), ~3 months post-dose two (V4), and ~9–10 months post-dose two (V5). Most participants were younger than 50 years old (70% of vaccinees) and identified as male and a member of the lesbian, gay, bisexual, transgender, and queer+ (LGBTQ+) community (77%), in line with the groups recommended to receive vaccination by the CDC (21). Of note, the median age for vaccinees with a history of smallpox vaccination was 62, compared to 33 for those without prior smallpox vaccination (referred to henceforth as naïve participants). Nearly a third of the participants had previously received a smallpox vaccination (referred to henceforth as experienced participants). Approximately half of those experienced participants were people with HIV (PWH), while only ~15% of naïve participants were PWH. During the 2022 outbreak, limitations in available doses led to an emergency use authorization (EUA) for a dose-sparing measure involving intradermal (ID) delivery of one-fifth of the dose used SC (22, 23). Although our study includes a range of dosing combinations: SC-SC, SC-ID, ID-SC, and ID-ID, SC-ID was the most common in the study (~33% of experienced participants and ~46% of naïve participants).

### MPXV H3- and A35-specific memory B cells are detectable at 1 year following MVA-BN vaccination

As previous generations of smallpox vaccines generated robust cellular memory responses, we first examined whether MVA-BN induced antigen-specific memory B cells at a late time point, V5 (Fig. 1). Representative MPXV proteins from the intracellular mature virion and extracellular enveloped virion forms were chosen, H3 and A35, respectively. Antigen-specific IgG+ memory B cells were assayed by ELISpot in MVA-BN vaccinees as well as MPXV convalescent subjects. The proportions of vaccinees with positive levels (i.e., greater than background) of memory B cells (MBCs) against H3 were 16% and 15% for naïve and experienced participants, respectively (Fig. 1B), and were 39% and 20% for A35, respectively (Fig. 1C). Representative well images are shown in Fig. 1D. These data indicate durable establishment of MPXV H3- and A35-specific memory B cells in both cohorts for at least a subset of participants in the study.

### Maximum MPXV neutralization titers are comparable for naïve and experienced MVA-BN vaccinees but differ in durability

**Fig 2:**
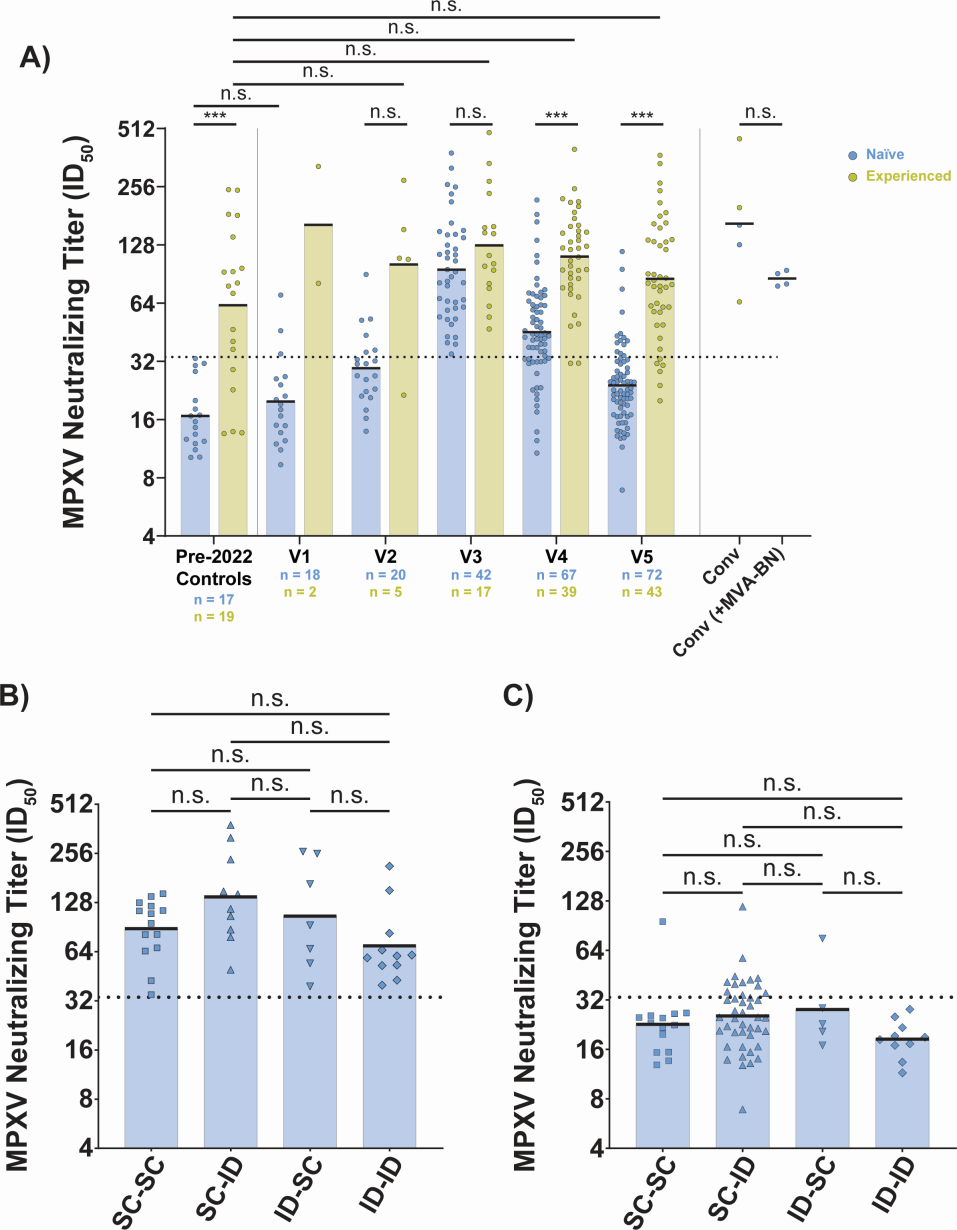
MPXV neutralizing titers are lower in naïve vaccinees compared to experienced individuals following MVA-BN vaccination, with no significant effect from the route of administration on titer. (**A**) Aggregate MPXV neutralizing titer data for each study visit sorted by prior smallpox vaccination status. Colored/black bars on each group indicate the geometric mean titer. Serum-neutralizing titers were measured by a fluorescence-based microneutralization assay using authentic clade IIb MPXV. Pre-2022 controls come from the NYU Langone Vaccine Center Biorepository. Convalescent (conv) participants are sorted by whether they received MVA-BN following MPXV infection; all samples are taken at ~1 year post-symptom onset. (**B**) Stratification of MPXV neutralization at V3 by route of administration in naïve vaccinees. (**C**) Stratification of MPXV neutralization at V5 by route of administration in naïve vaccinees. Horizontal, black dashed line in all panels indicates the positivity threshold for neutralization (ID_50_ = 33.7) as based on the pre-2022 negative controls (same method as Fig. 1). Statistical testing conducted by Kruskal-Wallis test with Dunn’s method for multiple comparisons for all panels.

While MPXV-specific memory B cell responses may mitigate severe disease, antibodies may play an important role in preventing infection, specifically neutralizing antibodies. To measure the neutralizing titers in the sera of OSMI participants, a fluorescence-based microneutralization assay was developed using authentic clade IIb MPXV. Pre-outbreak samples from experienced individuals had higher neutralizing titers at baseline compared to naïve people (Fig. 2A, pre-2022 controls) with no effect from the time since childhood vaccination on titers (Fig. S1), in line with prior work showing long-lasting durability for orthopoxvirus immunity (5–9). Aggregate data showed no statistically significant difference between naïve and experienced participants at V3 (geometric mean titers [GMTs] of 95 versus 127, respectively), highlighting similar maximum values for neutralizing titers (Fig. 2A). However, by V4, there was a statistically significant difference between the two groups (GMTs of 45 for naïve versus 111 for experienced). This difference persisted at V5 with 81% of naïve vaccinees below the positivity threshold, compared to only 14% of experienced vaccinees (GMTs of 24 versus 85, respectively). Nine mpox-convalescent individuals were included as another control group, some of whom had received at least one dose of MVA-BN (Table S1). At ~1 year after symptom onset, these participants had GMTs comparable to maximum titers (V3) for vaccinees, suggesting a more durable neutralizing antibody response post-MPXV infection as compared to post-MVA-BN vaccination. These trends in neutralizing antibodies were also observed for anti-MPXV H3 IgG binding antibody titers, as measured by ELISA, although a lower proportion of naïve MVA-BN vaccinees (50%) were below the limit of detection at V5 (Fig. S2A). Taken together, these data suggest that while MVA-BN induces appreciable neutralizing titers in all vaccinees, the durability of these antibodies is limited in naïve vaccinees.

### The dose-sparing ID route induces comparable peak titers in naïve vaccinees

The EUA for a dose-sparing ID route introduced several possible combinations of routes of administration, with SC-ID and ID-SC not having been previously studied. To address this question, we stratified MPXV neutralizing titers of naïve vaccinees by route of administration for V3 and V5 (Fig. 2B and C, respectively). From this analysis, we found no significant difference in peak titers (Fig. 2B) or durability of titers (Fig. 2C) across the different routes of administration. We next wanted to examine the usage of the smaller dose ID route within PWH, as prior studies of MVA-BN vaccination in PWH had examined only the approved SC route (24, 25). The enrolled participants with HIV all had CD4+ T cell counts over 250 cells/cmm and most were over 500 cells/cmm (Table S2). Combining all routes of vaccination, neutralizing titers were similar between HIV-uninfected participants and PWH in both the naïve and experienced groups (Fig. S3). When focusing only on those who received at least one ID dose, a similar result was found (Fig. S4). We additionally examined the effect of HIV status in a linear mixed-effects regression and similarly found no effect on neutralizing titers in naïve (Table S3) or experienced participants (Table S4). Lastly, using a linear mixed-effects model, we tested whether CD4 counts had an effect on neutralizing titers and found no such effect in naïve (Table S5) or experienced PWH (Table S6). These data indicate that the MVA-BN vaccine established similar humoral responses across the various combinations of routes of administration and that this finding holds within PWH with normal CD4 counts.

### Neutralizing titers decay faster in naïve vaccinees compared to experienced vaccinees

**Fig 3:**
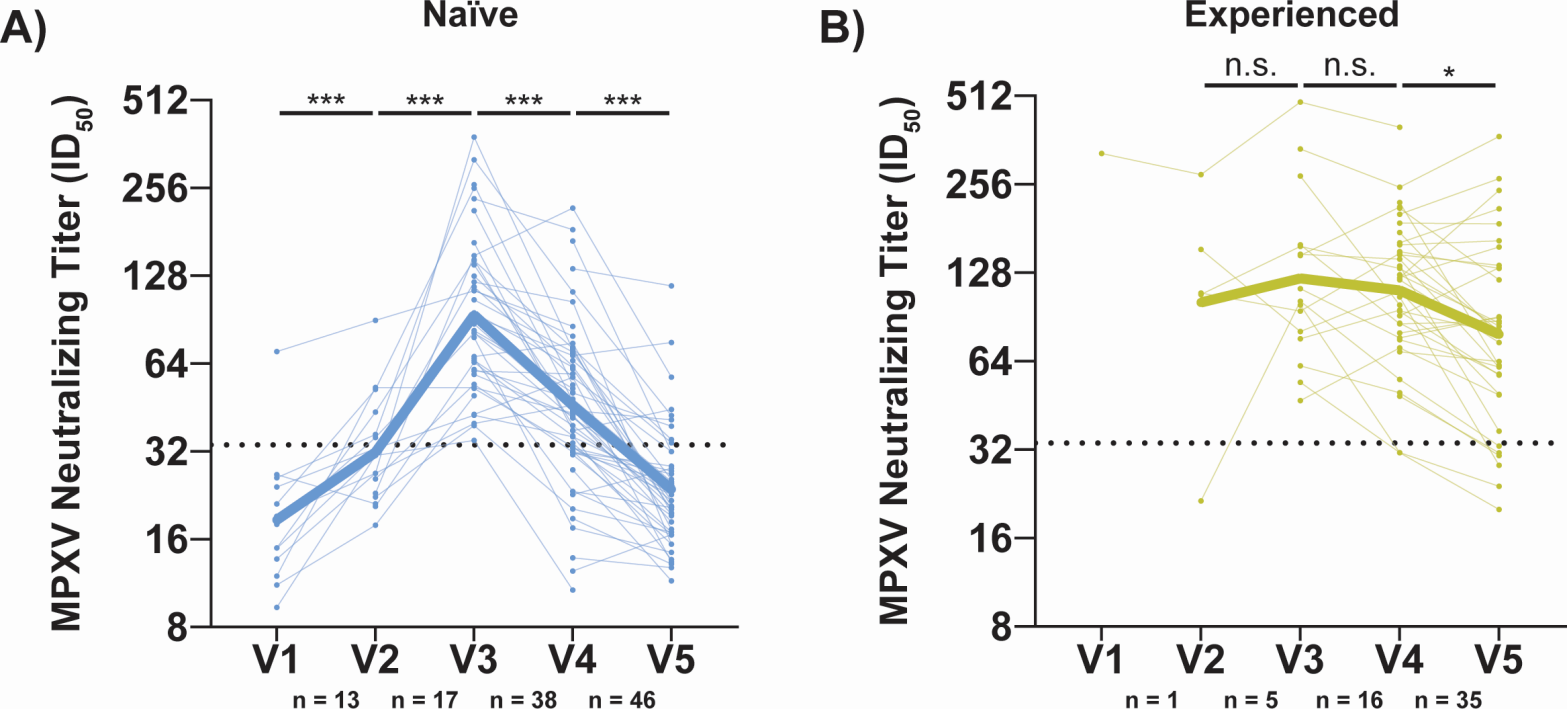
MPXV neutralizing titers begin to wane after 3 weeks post-second vaccination (**V3**) in naïve individuals but only after 3 months post-second vaccination (**V4**) in experienced vaccinees. (**A**) Longitudinal neutralizing titers from naïve participants across all five study visits. The bolded line indicates the mean neutralizing titer for participants at each time point. (**B**) Longitudinal neutralizing titers from experienced participants across all five study visits. The bolded line indicates the mean neutralizing titer for participants at each time point. Horizontal, black dashed line in all panels indicates the positivity threshold for neutralization (ID_50_ = 33.7) as based on the pre-2022 negative controls (same method as Fig. 1). Statistical testing conducted by Wilcoxon matched-pairs signed rank test. N below each panel indicates the number of visit-to-visit comparisons for a given pair of visits.

To further leverage the longitudinal nature of the OSMI study, we next examined participants with samples from consecutive visits using paired statistical analyses (Fig. 3). Following the first dose of MVA-BN, naïve participants already had higher neutralizing titers relative to pre-vaccination (Fig. 3A). Neutralizing titers peaked at V3 and declined thereafter. For experienced individuals (Fig. 3B), titers did not begin to decline until after V4. A similar pattern was seen with anti-MPXV H3 IgG titers (Fig. S2B and C). To evaluate the rate of change in each group, paired fold changes in MPXV neutralizing titers for V3 versus V4 and V4 versus V5 were compared between naïve and experienced individuals. In both instances, naïve participants had a greater fold change in titers from one visit to the next (Fig. S5).

**Fig 4:**
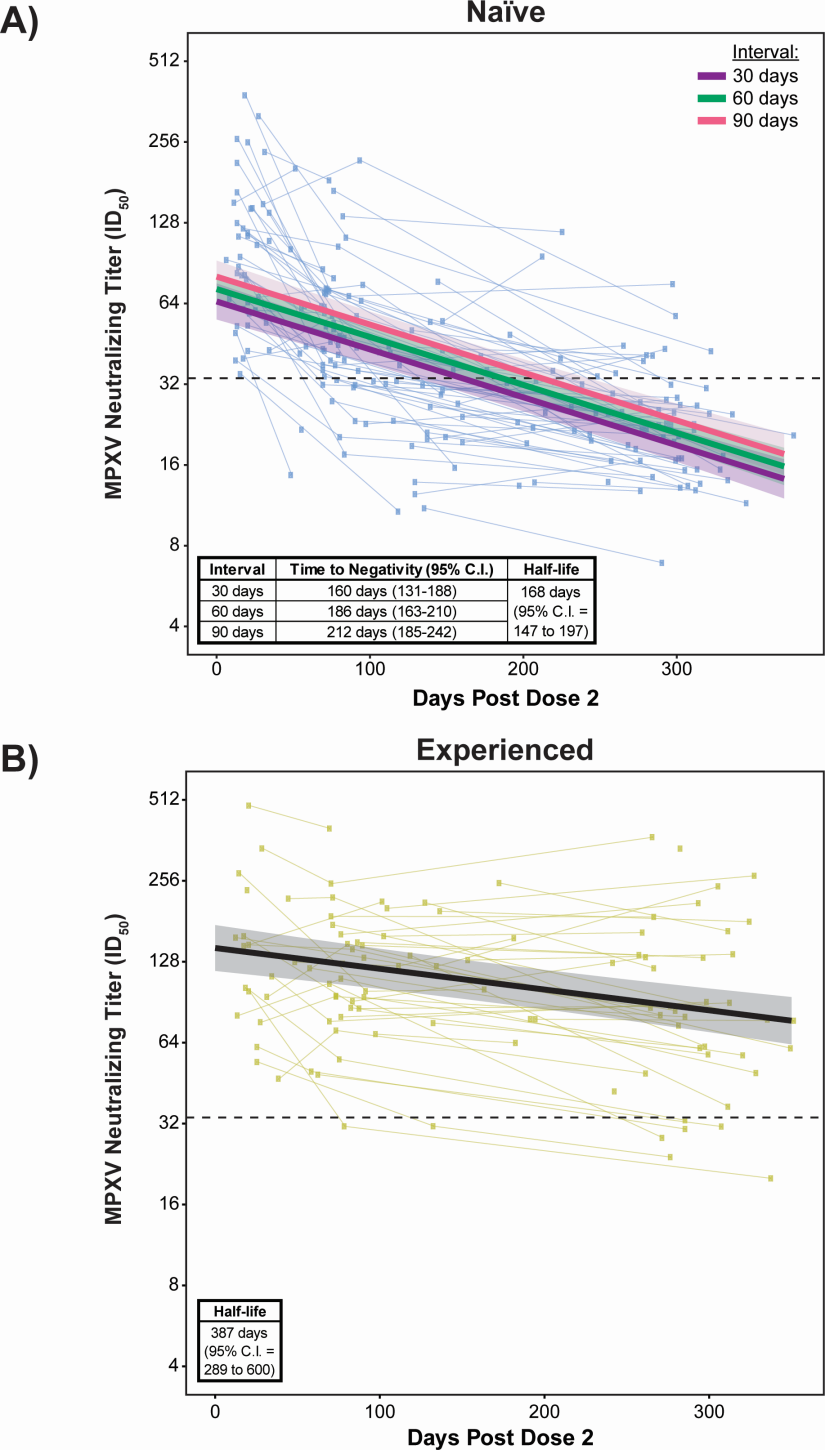
Modeling of neutralizing titer durability shows naïve individuals revert to negative titers by ~6 months, while experienced individuals remain positive beyond a year after MVA-BN vaccination. (**A**) Linear mixed-effects regression of naïve participants using all data points after the second dose of MVA-BN. Regressions of natural log-transformed neutralizing titers use days post-dose two and dosing interval as fixed effects and account for random subject effects by allowing for random intercepts. Colored regression lines with transparent ribbons indicate the predicted titers with a 95% confidence interval. Model parameters are listed in Table S7; 207 observations from 100 participants. (**B**) Linear mixed-effects regression of experienced participants using all data points after the second dose of MVA-BN. Regression of natural log-transformed neutralizing titers uses days post-dose two as a fixed effect and accounts for random subject effects by allowing for random intercepts. The solid black regression line with transparent ribbon indicates the predicted titers with 95% confidence interval. Model parameters are listed in Table S8; 106 observations from 48 participants. Horizontal, black dashed line in each panel indicates the positivity threshold for neutralization (Fig. 2).

We then assessed this observation with a statistical model, using linear mixed-effects regression (Table S3 and S4). For naïve participants, neutralizing titers were associated with dosing interval and days post-dose two (model parameters can be found in Table S7), while neutralizing titers in experienced participants were only associated with days post-dose two (Table S8). Using these models, the half-life of neutralizing antibodies was predicted to be 168 days (95% CI: 147 to 197 days) in naïve individuals, regardless of dosing interval. Participants who received the 1-month dosing interval, which is the minimum recommended interval, lost titer seropositivity by 160 days post-dose two (Fig. 4A). The time to negativity increased by ~1 month for each additional month between MVA-BN doses (Fig. 4A, inset table). This was likely driven, in part, by the positive correlation of peak MPXV neutralizing titers with dosing interval (Fig. S6). Antibody decline was far slower in experienced MVA-BN vaccines (Fig. 4B) with a predicted half-life of 387 days (95% CI: 289 to 600 days). The predicted time to cross the seropositivity threshold was >350 days. Together, these data highlight the difference in the longevity of serum neutralization activity for the two-dose MVA-BN regimen in naïve people versus experienced people.

### IgG responses to MPXV A35 and H3 are prevalent regardless of smallpox vaccination history but have lower avidity in naïve vaccinees

To further characterize the serological responses of the naïve and experienced groups, a subset of participants from each group was chosen based on those that consistently participated from V3 to V5. These subsets had neutralizing titers similar to the full cohort (Fig. S7). Using a multiplexed bead-based immunoassay, IgG binding titers and avidity for eight MPXV antigens (Table 2) were measured: A29, A30, A35, B16, B21, E8, H3, and L1. These antigens were selected as known protective targets (26–33) or potential neutralizing targets based on previous reports (34–36). IgG avidity was measured in this assay using an additional incubation with 2 M ammonium thiocyanate following serum incubation. We found that the H3 and A35 viral proteins were immunogenic in both naïve and experienced individuals across all time points post-dose two (Fig. S8A). Binding titers for H3 and A35 were also correlated with MPXV neutralization in both groups across multiple time points (Fig. S8B). As the VACV homologs of H3 and A35 have been previously shown to be protective antibody targets in orthopoxvirus infections (26–28) and our other antigens showed less positive signal, we decided to focus on these two antigens for our analysis.

**TABLE 2:** Eight MPXV proteins used in the multiplexed immunoassay for antibody binding and avidity: their location in virions and function in viral infection

MPXV protein	VACV Copenhagen homolog	Function/location	References
H3	H3	Intracellular mature virus (IMV) protein, binds heparan	(26, 27)
A35	A33	Extracellular enveloped virus protein, envelope glycoprotein	(28, 32, 33)
E8	D8	IMV protein, binds chondroitin	(29)
B16	B19	Type I interferon antagonist	(37)
A29	A27	IMV protein, binds heparan	(30, 32)
A30	A28	Member of entry-fusion complex	(31)
B21	N/A	Presumed surface protein	(36)
L1	J1	IMV protein, virion morphogenesis	(35, 38)

**Fig 5:**
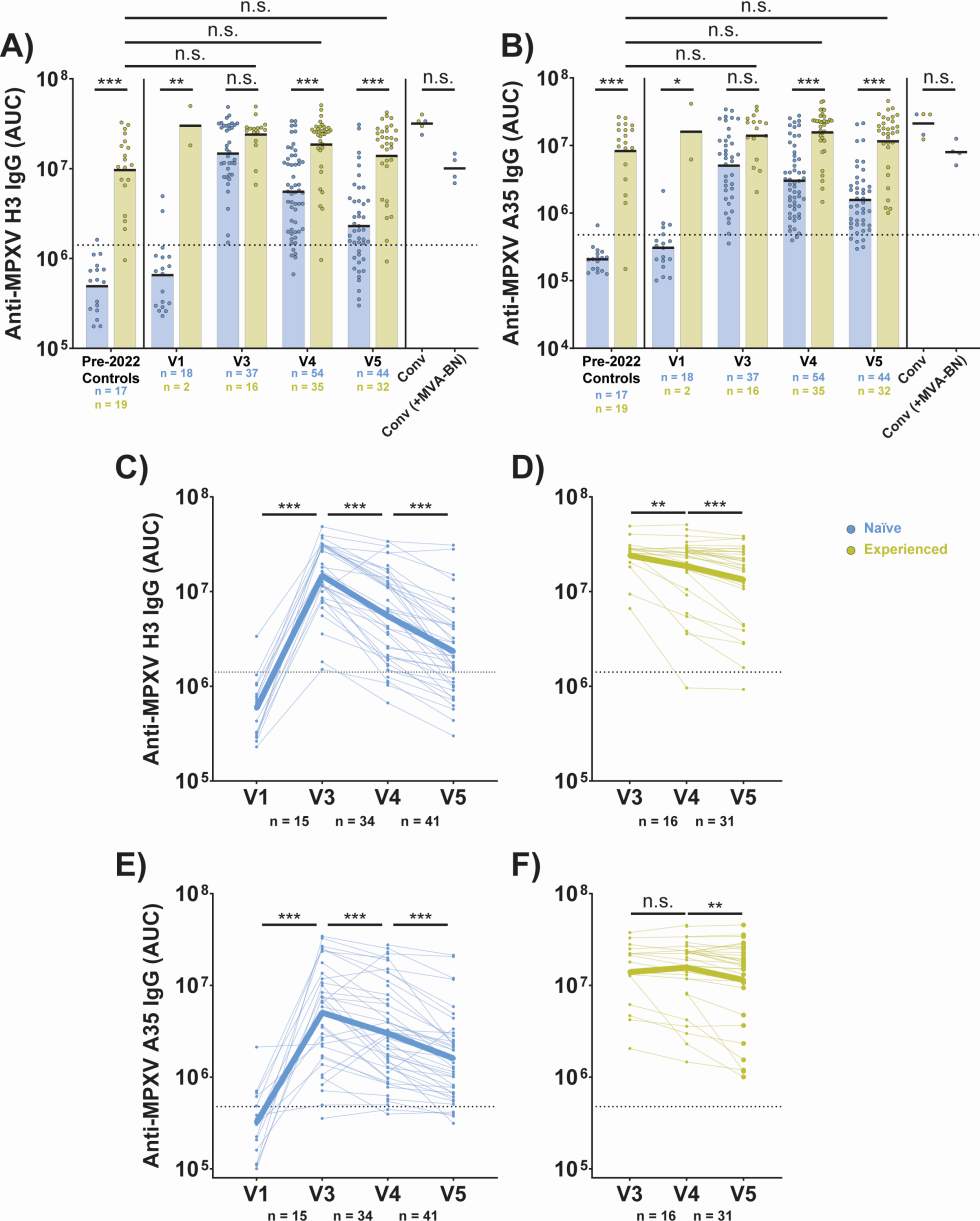
Anti-MPXV H3 and -A35 IgG reach comparable peak titers in naïve and experienced participants, but wane more quickly in naïve participants following MVA-BN vaccination. (**A**) Aggregate anti-MPXV H3 IgG titers as measured by multiplexed immunoassay. Titers are reported as AUC from two serum dilutions. (**B**) Aggregate anti-MPXV A35 IgG titers as measured by multiplexed immunoassay. Titers are reported as AUC from two serum dilutions. (**C**) Longitudinal anti-MPXV H3 IgG titers for naïve participants at V1 and V3–5 as measured by multiplexed immunoassay. Titers are reported as AUC from two serum dilutions. (**D**) Same as C, but for experienced participants across V3–5. (**E**) Longitudinal anti-MPXV A35 IgG titers for naïve participants at V1 and V3–5 as measured by multiplexed immunoassay. Titers are reported as AUC from two serum dilutions. (**F**) Same as E, but for experienced participants across V3–5. MPXV convalescent (conv) samples were all taken at ~1 year post-symptom onset. In panels A and B, colored/black bars indicate the geometric mean. For panels C–F, the bolded line indicates the mean IgG titer for participants at each time point. Black, dashed line in all panels is the positivity threshold for binding as determined by pre-2022 negative controls (same samples and method as in Fig. 2). Statistical testing conducted by Kruskal-Wallis test with Dunn’s method for multiple comparisons for panels A and B, and by Wilcoxon matched-pairs signed rank test for panels C–F.

Like MPXV neutralizing titers, anti-H3 (Fig. 5A) and anti-A35 (Fig. 5B) IgG titers reached comparable maximum titers in naïve and experienced individuals. IgG titers against each antigen also declined faster in naïve participants as compared to experienced vaccinees. Anti-H3 IgG began to wane after V3 in both groups (Fig. 5C and D), while anti-A35 IgG began to wane after V3 in naïve individuals (Fig. 5E), but only after V4 in experienced individuals (Fig. 5F). The timing of A35 waning more closely matched the waning of neutralizing titers in experienced vaccinees.

**Fig 6:**
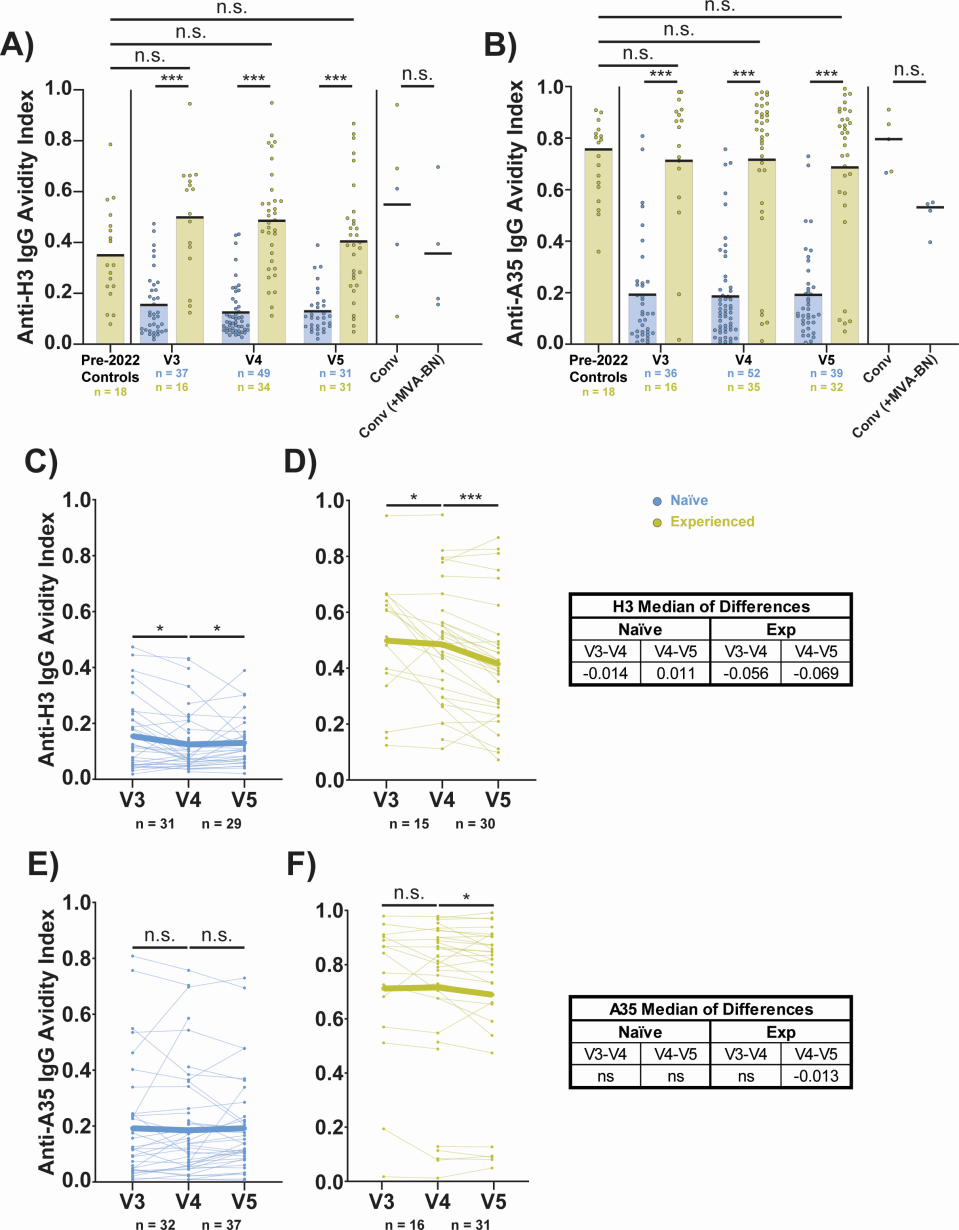
Naïve vaccinees generate low avidity IgG against MPXV H3 and A35 after vaccination with MVA-BN. (**A**) Aggregate avidity indices for anti-MPXV H3 IgG. (**B**) Aggregate avidity indices for anti-MPXV A35 IgG. (**C**) Longitudinal avidity indices for anti-MPXV H3 IgG antibodies for V3–5 of naïve participants. (**D**) Same as C, but for experienced participants. (**E**) Longitudinal avidity indices for anti-MPXV A35 IgG antibodies for V3–5 of naïve participants. (**F**) Same as E, but for experienced participants. Avidity was measured in a multiplexed immunoassay using a 2 M ammonium thiocyanate (NH_4_SCN) wash and expressed as the ratio of the AUC from the NH_4_SCN condition over the AUC from a PBS control condition. Convalescent (conv) samples were all taken at ~1 year post-mpox symptom onset. In panels A and B, colored/black bars indicate the mean. Statistical testing conducted by Kruskal-Wallis test with Dunn’s method for multiple comparisons for panels A and B, and by Wilcoxon matched-pairs signed rank test for panels C–F. Exp, experienced vaccinees (prior history of smallpox vaccination).

We also measured avidity for anti-H3 and anti-A35 IgG. Aggregate data showed higher IgG avidity in the experienced group for the H3 (Fig. 6A) and A35 proteins (Fig. 6B) across all time points post-dose two. Surprisingly, naïve vaccinees had longitudinal changes in anti-H3 IgG avidity that, while statistically significant, were low and inconsistent across time (Fig. 6C). However, anti-H3 IgG avidity declined from V3 to V4 and V4 to V5 in the experienced group (Fig. 6D), perhaps indicating the generation of *de novo* low avidity responses that reduce the bulk avidity in the sera. Changes in the avidity of anti-A35 IgG were more subdued but in a similar direction (Fig. 6E and F). Taken together, these data indicate similar B cell antigenic targets for naïve and experienced MVA-BN vaccinees, but with avidity being much higher in experienced MVA-BN vaccinees, which may imply limited affinity maturation in naïve individuals.

## DISCUSSION

We report here an analysis through 1 year of the MPXV-specific serum antibody magnitude, specificity, neutralizing function, avidity, and blood MBCs following the two-dose MVA-BN vaccination regimen. The current data indicate that IgG antibodies induced by MVA-BN are limited in their durability in naïve adults. We identified two MPXV surface proteins, H3 and A35, that were strongly targeted by IgG after MVA-BN administration. We found that the IgG response to those proteins was low avidity in naïve MVA-BN recipients in comparison to experienced MVA-BN recipients. We also demonstrated that H3- and A35-specific MBCs are detectable in circulation in subsets of vaccinees 1 year after vaccination despite the limited durability of circulating MPXV-specific antibodies.

The observed differences between naïve and experienced MVA-BN vaccinees may have several potential causes. While the experienced group had previously received a dose of a replicating vaccine (vaccinia), the naïve group had received only the nonreplicating MVA-BN. The difference in the initial poxvirus vaccination platform is likely to generate different inflammatory states, which we have recently shown durably impacts the CD4+ T cell memory response (39). The two groups also differ in the quantity of total vaccine doses (three for experienced versus two for naïve). To this point, it is possible that an additional dose of MVA-BN would put the naïve group at parity with the experienced group, which should be investigated with booster studies. Lastly, the timing of vaccination varies between the two groups, with smallpox vaccination generally taking place in childhood as opposed to mpox vaccination in adulthood. Prior work has shown that first-time vaccination with first- or second-generation replicating VACV vaccines in adulthood yields more durable neutralizing titers than were observed here in naïve adults receiving MVA-BN (~10 years versus ~6 months, respectively) (9). Taken together, these data would argue that VACV vaccine replication is a significant contributor to the durability of circulating antibodies.

Beyond the impact of prior smallpox vaccination on MVA-BN responses, we observe an association between dosing interval and neutralizing titer durability. We previously reported no effect of dosing interval on anti-MPXV H3 IgG titers in naïve individuals (14), which could imply differences in humoral responses for anti-H3 IgG binding titers and orthopoxvirus neutralization (40, 41). As multiple protein targets of neutralization are available for immune responses, it is likely that redundancies in antibody responses lead to imperfect correlations between binding and neutralizing responses. The delayed rollout of second doses during the 2022 outbreak offered a unique opportunity to begin studying varied dosing schedules, but controlled studies will be necessary to identify an ideal dose interval and whether the relationship between dosing interval and titer durability remains linear beyond 3 months.

It is interesting to note that there was no effect of interval on neutralizing titers in experienced individuals. A possible explanation supported by previous work (19, 42) is that the second dose of MVA-BN provides no further immune boost in this group. Our own work using a pre-2022 cohort of smallpox-vaccinated individuals shows that while no post-vaccination timepoint is significantly different from the control group, there is a clear trend toward higher neutralizing titers after the second dose of vaccination. This trend of immune boost from two doses is not evident when using paired data points, the more robust approach, from post-dose one to post-dose two in the experienced group. However, we observed significant waning after V4, suggesting an effect from MVA-BN. These data support the need for studies specifically focused on the MPXV-specific immunological benefits of MVA-BN vaccination in individuals with prior smallpox vaccination.

While we did find modest proportions of vaccinees with H3- and A35-specific MBCs in circulation 1 year after vaccination, these proportions were much lower than the positivity rates of corresponding antibodies for each. As MBCs can also reside in tissues, such as the lymph node and spleen, this could diminish their availability for sampling via peripheral blood. Furthermore, we saw no difference in MBCs between naïve and experienced vaccinees despite such a difference existing for antibody titers. As plasma cells (PCs) are the actual producers of antibodies, it is possible that there is a disconnect between PC formation and the establishment of an MBC reservoir as these two populations are distinct. One possible mechanism of this disconnect would be induction by MVA-BN of a primarily extrafollicular B cell maturation pathway, which is thought to favor PC formation (43). An extrafollicular path of B cell maturation could also explain the absence of IgG avidity maturation seen over time in naïve vaccinees. Further interrogation of the bone marrow compartment and germinal centers following MVA-BN vaccination is needed to better understand how PCs and MBCs are being formed. Such studies will also clarify what long-term protection is offered by MVA-BN vaccination. Current data in the field highlight the importance of neutralizing antibodies for protection from infection using animal models of MPXV (44, 45), but human data is more limited. Our understanding of protection duration within humans is primarily based upon CDC data showing that the median time between receipt of MVA-BN dose two and a breakthrough MPXV infection is 266 days (46), which falls just a few months beyond our estimate of when naïve vaccinees lose serum neutralization titers. Which immune responses offer protection beyond this point requires further investigation.

These data are particularly timely considering the ongoing mpox clade I outbreak in the Democratic Republic of Congo (47, 48). As vaccination remains severely limited in the DRC and neighboring nations, our data would support further clinical evaluation of several dose-sparing approaches such as a 1/5 ID dose as well as delayed second doses. This clade I outbreak is also a critical opportunity to understand how MPXV immunity may differ between the two clades. As the two clades share ~95% sequence identity (49), it is possible that immunity is highly conserved.

As an observational study, OSMI has limitations. The emergent 2022 mpox outbreak limited the collection of baseline samples for many of the participants in OSMI, especially the smaller group of individuals with prior smallpox vaccination. The speed of the public health emergency also created difficulties in defining narrow time windows for each study visit, as would be done in a typical vaccine clinical trial. In addition, childhood records of smallpox vaccination were incomplete and did not account for regional variations in cessation of childhood smallpox vaccination within the United States. Beyond the limitations of observational studies on patient recruitment and classification, we focus here only on the B cell side of adaptive immunity. Future efforts should consider cellular immunity more broadly when studying differences established by replicating versus nonreplicating vaccines. In addition, the study of antibody responses against MPXV proteins beyond those examined here may offer further insights into important neutralizing targets. Other work in the field has already highlighted the MPXV B2, B6, and M1 proteins as other immunodominant proteins following MVA-BN vaccination (13), offering an additional array of proteins of interest.

Directions for future work include further study of the magnitudes, specificities, qualities, and durability of memory B and T cells following the two-dose MVA-BN regimen. Our work demonstrates that MPXV-specific MBCs can remain in circulation for at least a year following MVA-BN vaccination. However, a critical unanswered question is whether the B cell memory responses induced by the MVA-BN two-dose regimen in naïve vaccinees are sufficient to protect against mpox disease when circulating neutralizing antibodies are no longer detectable. Further studies of breakthrough infections, booster doses, and correlates of protection are needed to better answer these questions. To this end, human challenge studies using VACV-based vaccination following MVA-BN vaccination would offer a tractable framework for beginning to examine these issues (50). In addition, the immunity induced by MPXV infection will need further characterization to determine whether subsequent vaccination is needed for durable, protective immunity.

Overall, we have shown that the two-dose MVA-BN regimen in naïve individuals produces low avidity, nondurable serological responses, in contrast to the response to smallpox vaccination, which generates MPXV-specific antibody responses that are durable and high avidity. An ideal future mpox vaccine would combine the safety advantages of the nonreplicating MVA-BN vaccine with the robust, durable protective immunity of replicating VACV vaccines. Future studies of MVA-BN cellular immunity may offer clues as to how to improve immune responses, whether that is through adjuvant, additional doses, higher doses, altered dosing intervals, or even virologic changes to MVA-BN itself. This is particularly important as mpox outbreaks look poised to continue. We believe that these discoveries into antibody specificity, quality, and durability offer crucial insights for the development of next-generation mpox-specific vaccines (44, 45, 51–53).

## Data Availability

All data are available in the supplemental material. Raw images from the ELISpot assay are available upon request.

## SUPPLEMENTAL MATERIAL

Data Set S1 jvi.00253-25-s0001.xlsx All raw data used for main figures.

Data Set S2 jvi.00253-25-s0002.xlsx All raw data used for supplemental figures.

Supplemental material jvi.00253-25-s0003.pdf Tables S1 to S8; Fig. S1 to S8.

ASM does not own the copyrights to Supplemental Material that may be linked to, or accessed through, an article. The authors have granted ASM a non-exclusive, world-wide license to publish the Supplemental Material files. Please contact the corresponding author directly for reuse.
