# Correction for Oom et al., “The two-dose MVA-BN mpox vaccine induces a nondurable and low avidity MPXV-specific antibody response”

**DOI:** 10.1128/jvi.00872-25

**Published:** 2025-09-04

**Authors:** Aaron L. Oom, Kesi K. Wilson, Miilani Yonatan, Stephanie Rettig, Heekoung Allison Youn, Michael Tuen, Yusra Shah, Ashley L. DuMont, Hayley M. Belli, Jane R. Zucker, Jennifer B. Rosen, Ramin Sedaghat Herati, Marie I. Samanovic, Ralf Duerr, Angelica C. Kottkamp, Mark J. Mulligan, on behalf of the NYC OSMI Study Group

## AUTHOR CORRECTION

Volume 99, no. 9, e00253-25, 2025, https://doi.org/10.1128/jvi.00253-25. Due to an equipment malfunction, the serum samples included in the published article were improperly heat-inactivated at 43°C as opposed to the standard 56°C. To address this, tests of different heat inactivation (HI) conditions were conducted for both our neutralization and binding assays using a variety of samples from across our cohort. We found that proper heat inactivation significantly reduces MPXV neutralization, while the addition of 5% guinea pig serum (GPS, Sigma-Aldrich G9774) restores signal (Fig. 7A). This is in line with the finding from Hubert et al. that most of the MPXV neutralizing capacity is dependent upon complement (M. Hubert, F. Guivel-Benhassine, T. Bruel, F. Porrot, et al., 2023, Cell Host Microbe 31:937-948, https://doi.org/10.1016/j.chom.2023.05.001); this observation was described by Hubert and colleagues in samples from MVA-BN-vaccinated individuals (with and without prior smallpox-vaccination), smallpox-vaccinated individuals, and MPXV convalescent patients. We saw no significant effect of proper heat inactivation on binding (Fig. 7B and C). However, for samples lower in the assay’s dynamic range, the effect appeared larger. These data clearly indicate that samples heated at 43°C in our original article behave similarly to samples that were not heat inactivated.

To determine whether the findings reported in our article would remain when samples were properly heat inactivated, we re-tested samples from the MVA-BN only group (naïve vaccinees), MVA-BN +smallpox vaccine group (experienced vaccinees), and the smallpox vaccine-only group (experienced controls). For MPXV neutralization, we found a significant correlation between the values previously published using heat treatment at 43°C and the values obtained using proper heat inactivation (56°C) with added GPS (Fig. 7D). Furthermore, when we assessed IgG binding to the MPXV H3 and A35 proteins with heat-inactivated (56°C) and non-heat-inactivated samples (43°C), we again observed a significant correlation (Fig. 7E and F). Taken together, these data clearly indicate that non-heat-inactivated samples behave similarly to (i) heat-inactivated samples for IgG binding and (ii) heat-inactivated samples with added GPS for neutralization. As such, we believe that our original findings stand. We apologize for this error and any inconvenience it might have caused.

**Fig 7:**
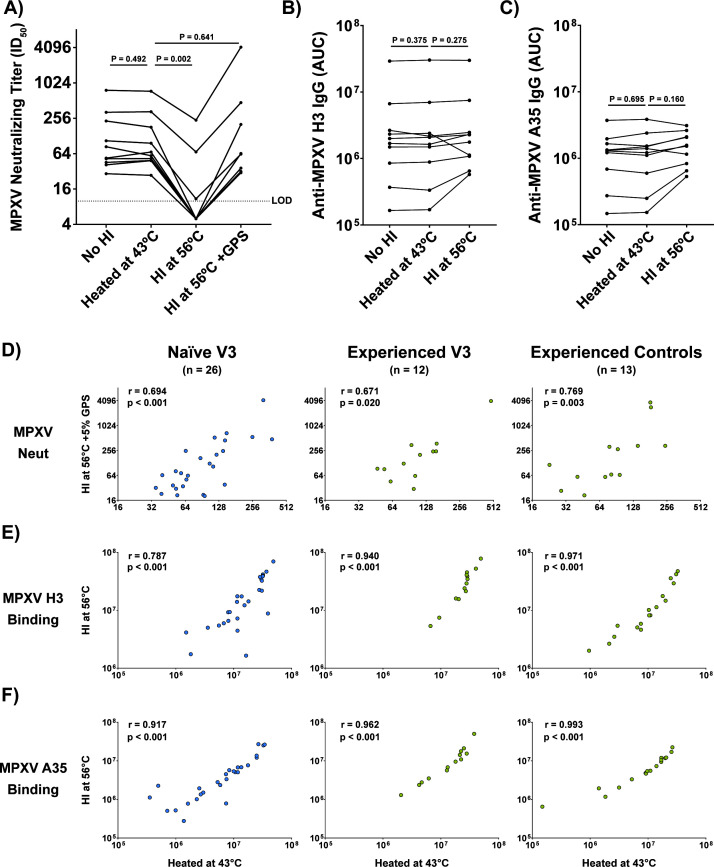
Heat treatment at 43°C yields binding and neutralizing titers that significantly correlate with values from heat inactivation at 56°C (with added GPS for neutralization). (**A**) Samples from V3 were tested for MPXV neutralization using the method described in our published article. Guinea pig serum (GPS, 5%) was used as a source of complement. Samples with an ID_50_ of less than 10 are displayed as an ID_50_ value of 5. (**B** and **C**) Samples from V5 were assayed for either anti-MPXV H3 IgG (**B**) or anti-MPXV A35 IgG (**C**) using the multiplexed immunoassay described in our published article. (**D**) Spearman correlation of MPXV neutralization with heat treatment at 43°C or heat inactivation at 56°C with added 5% GPS. (**E**) Spearman correlation of anti-MPXV H3 IgG titers with heat treatment at 43°C or heat inactivation at 56°C. (**F**) Spearman correlation of anti-MPXV A35 IgG titers with heat treatment at 43°C or heat inactivation at 56°C. Statistics for panels A through C were conducted using the Wilcoxon matched-pairs signed rank test.

## EDITOR'S NOTE

**Ed. Note**: This correction was reviewed by the original article's peer reviewers and approved in consultation with the handling editor and journal editors-in-chief.

