# Supplementary material for: The two-dose MVA-BN mpox vaccine induces a nondurable and low avidity MPXV-specific antibody response
Source: J Virol. 2025 Mar 31;99(9):e00253-25. doi: 10.1128/jvi.00253-25 (PMC12456018; doi:10.1128/jvi.00253-25)
Supplement: Supplemental material — Tables S1 to S8; Fig. S1 to S8. [file jvi.00253-25-s0003.pdf]

Supplemental Materials:
The Two-dose MVA-BN Mpox Vaccine Induces a Nondurable and Low Avidity MPXV-specific Antibody Response

Aaron L. Oom, Kesi K. Wilson, Miilani Yonatan, Stephanie Rettig, Heekoung Allison Youn, Michael Tuen, Yusra Shah, Ashley L. DuMont, Hayley M. Belli, Jane R. Zucker, Jennifer B. Rosen, Ramin Sedaghat Herati, Marie I. Samanovic, Ralf Duerr, Angelica C. Kottkamp, and Mark J. Mulligan on behalf of the NYC OSMI Study Group

Table S1: Sample information for convalescent participants.

Participant	Age Range	Prior Smallpox Vaccination	HIV Status	Received Tecovirimat?	Dose 1 DPO	Dose 1 Route	Dose 2 DPO	Dose 2 Route	Sample DPO
Conv 1	40-50	No	PWH	Yes	-25	SC	46	ID	340
Conv 2	30-40	No	HIV uninfected	No	9	SC	87	ID	336
Conv 3	20-30	No	HIV uninfected	Yes	-1	SC			386
Conv 4	40-50	No	PWH	Yes	78	ID			388
Conv 5	30-40	No	PWH	Yes					335
Conv 6	50-60	Yes	PWH	Yes					338
Conv 7	60-70	Yes	PWH	No					391
Conv 8	60-70	Yes	PWH	No					433
Conv 9	30-40	No	PWH	No					386

Participants above the horizontal line received one or two doses of MVA-BN.
Conv, convalescent; PWH, person with HIV; SC, subcutaneous; ID, intradermal; DPO, days post-symptom onset.

Table S2: CD4 counts at time of enrollment for OSMI participants with HIV.

Naïve CD4 Counts	Exp CD4 Counts
267	284
321	350
347	351
491	438
500.5	450
574.5	482.5
576	538.5
648	552
744	600.5
750	675
797	680
842	680
907	685
1387	703
1500	751.5
	755
	760
	859
	876
	890
	902
	948
	996
	1069
	1124

Exp, prior smallpox vaccination.

Table S3: Linear mixed-effects model parameters for naïve participants using days post dose 2 (DPV2), age, HIV status, route of administration, and dosing interval as fixed effects and subject (OSMI) as a random effect; age is no longer significant following the removal of HIV status and route of administration.

**log(Neut)**			
Predictors	*Estimates*	*CI*	*p*
(Intercept)	4.4587	3.9333 – 4.9842	**<0.0001**
DPV2	-0.0041	-0.0047 – -0.0035	**<0.0001**
Age	-0.0146	-0.0281 – -0.0011	**0.0347**
HIV [Pos]	0.0068	-0.2609 – 0.2745	0.9599
Route [ID-SC]	0.3527	-0.0468 – 0.7522	0.0833
Route [SC-ID]	0.1622	-0.0967 – 0.4211	0.2181
Route [SC-SC]	0.1504	-0.1429 – 0.4438	0.3131
Interval	0.0030	0.0008 – 0.0052	**0.0084**
**Random Effects**			
σ^2	0.17		
$\tau_{00\ OSMI}$	0.13		
ICC	0.43		
N_{OSMI}	100		
Observations	207		
Marginal R^2 / Conditional R^2	0.467 / 0.694		
REML Criterion at Convergence	354.5		

Table S4: Linear mixed-effects model parameters for experienced participants using days post dose 2 (DPV2), dosing interval, route of administration, age, and HIV status as fixed effects and subject (OSMI) as a random effect.

ln(Neut)			
Predictors	*Estimates*	*CI*	*p*
(Intercept)	4.1590	2.3366 – 5.9814	**<0.0001**
DPV2	-0.0018	-0.0024 – -0.0011	**<0.0001**
Age	0.0142	-0.0081 – 0.0366	0.2091
HIV [Pos]	0.1068	-0.2439 – 0.4575	0.5469
Route [ID-ID]	-0.0481	-1.3567 – 1.2605	0.9420
Route [ID-SC]	-0.4648	-1.8034 – 0.8739	0.4923
Route [SC-ID]	-0.1868	-1.4623 – 1.0886	0.7718
Route [SC-SC]	-0.2444	-1.5097 – 1.0209	0.7023
Interval	0.0011	-0.0032 – 0.0054	0.6144
**Random Effects**			
σ^2	0.11		
$\tau_{00\ OSMI}$	0.30		
ICC	0.74		
N_{OSMI}	48		
Observations	106		
Marginal R^2 / Conditional R^2	0.153 / 0.779		
REML Criterion at Convergence	187.6		

Table S5: Linear mixed-effects model parameters for naïve PWH using days post dose 2 (DPV2) and CD4 counts as fixed effects and subject (OSMI) as a random effect.

ln(Neut)			
Predictors	*Estimates*	*CI*	*p*
(Intercept)	4.4747	4.0205 – 4.9288	**<0.0001**
DPV2	-0.0036	-0.0049 – -0.0023	**<0.0001**
CD4	-0.0003	-0.0008 – 0.0002	0.2310
**Random Effects**			
σ^2	0.10		
$\tau_{00\ OSMI}$	0.05		
ICC	0.36		
N_{OSMI}	14		
Observations	29		
Marginal R^2 / Conditional R^2	0.532 / 0.700		
REML Criterion at Convergence	53.3		

Table S6: Linear mixed-effects model parameters for experienced PWH using days post dose 2 (DPV2) and CD4 counts as fixed effects and subject (OSMI) as a random effect.

**ln(Neut)**			
Predictors	*Estimates*	*CI*	*p*
(Intercept)	4.5443	3.6179 – 5.4707	**<0.0001**
DPV2	-0.0016	-0.0025 – -0.0008	**0.0004**
CD4	0.0006	-0.0006 – 0.0019	0.3100
**Random Effects**			
σ^2	0.08		
$\tau_{00\ OSMI}$	0.44		
ICC	0.84		
N_{OSMI}	25		
Observations	52		
Marginal R^2 / Conditional R^2	0.095 / 0.856		
REML Criterion at Convergence	105.1		

Table S7: Linear mixed-effects model parameters for naïve participants using days post dose 2 (DPV2) and dosing interval as fixed effects and subject (OSMI) as a random effect.

ln(Neut)			
Predictors	*Estimates*	*CI*	*p*
(Intercept)	4.0699	3.8736 – 4.2662	**<0.0001**
DPV2	-0.0041	-0.0047 – -0.0035	**<0.0001**
Interval	0.0036	0.0014 – 0.0057	**0.0011**
**Random Effects**			
σ^2	0.17		
$\tau_{00\ OSMI}$	0.13		
ICC	0.44		
N_{OSMI}	100		
Observations	207		
Marginal R^2 / Conditional R^2	0.449 / 0.692		
REML Criterion at Convergence	344.6		

Table S8: Linear mixed-effects model parameters for experienced participants using days post dose 2 (DPV2) as a fixed effect and subject (OSMI) as a random effect.

ln(Neut)			
Predictors	*Estimates*	*CI*	*p*
(Intercept)	4.9694	4.7733 – 5.1656	**<0.0001**
DPV2	-0.0018	-0.0024 – -0.0012	**<0.0001**
**Random Effects**			
σ^2	0.11		
$\tau_{00\ OSMI}$	0.29		
ICC	0.73		
N_{OSMI}	48		
Observations	106		
Marginal R^2 / Conditional R^2	0.091 / 0.754		
REML Criterion at Convergence	170.2		

Figure S1

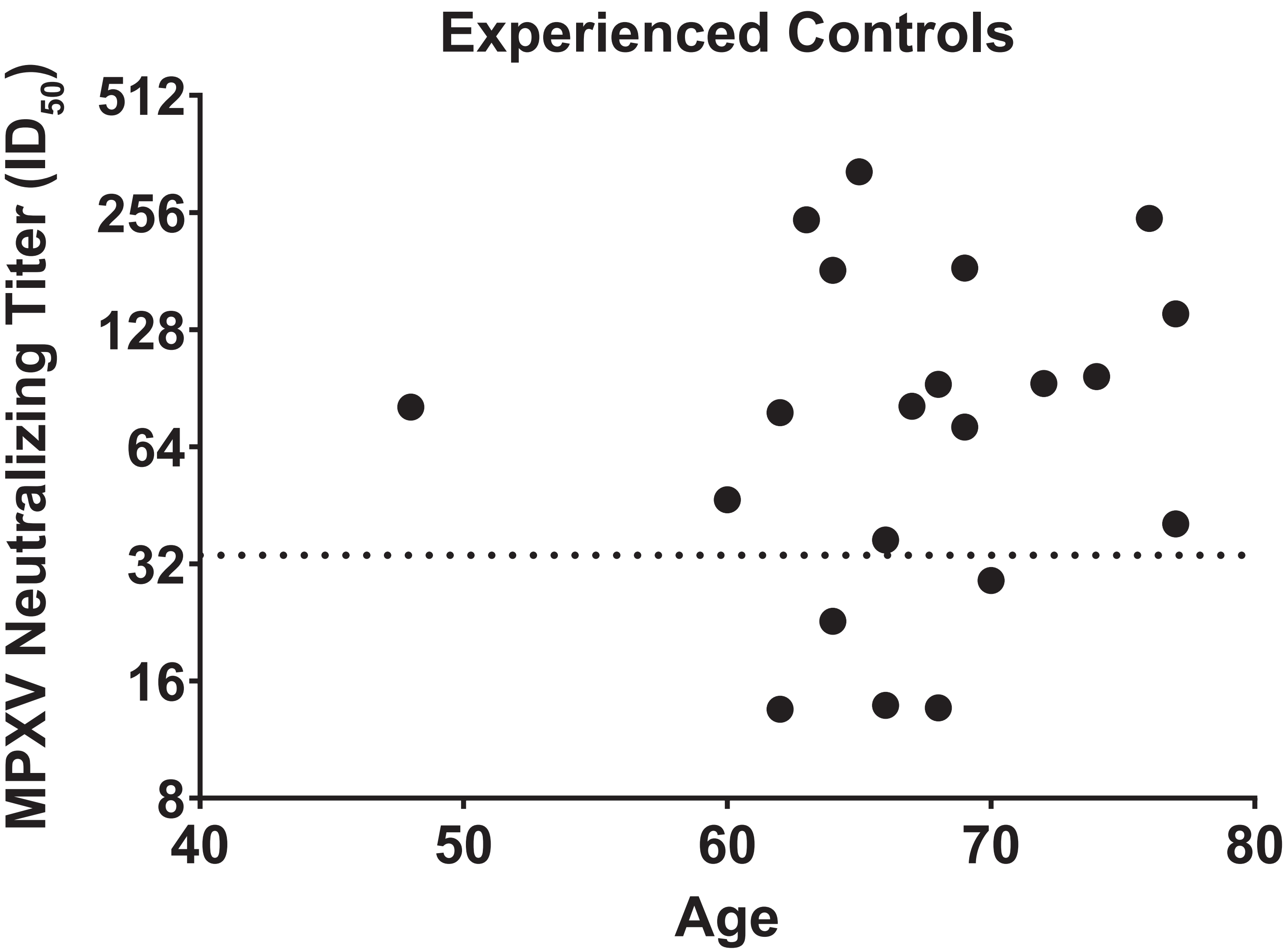
Experienced Controls
MPXV Neutralizing Titer (ID_{50})
512
256
128
64
32
16
8
40
50
60
70
80
Age

Figure S1: Time since childhood smallpox vaccination has no impact on MPXV neutralization. MPXV neutralizing titers as measured by microneutralization assay with authentic clade IIb virus for pre-2022 serum samples in individuals with childhood smallpox vaccination. Dashed line represents threshold for seropositivity as discussed in Fig. 2. Neither linear (Pearson) nor non-linear (Spearman) correlations achieved statistical significance.

Figure S2

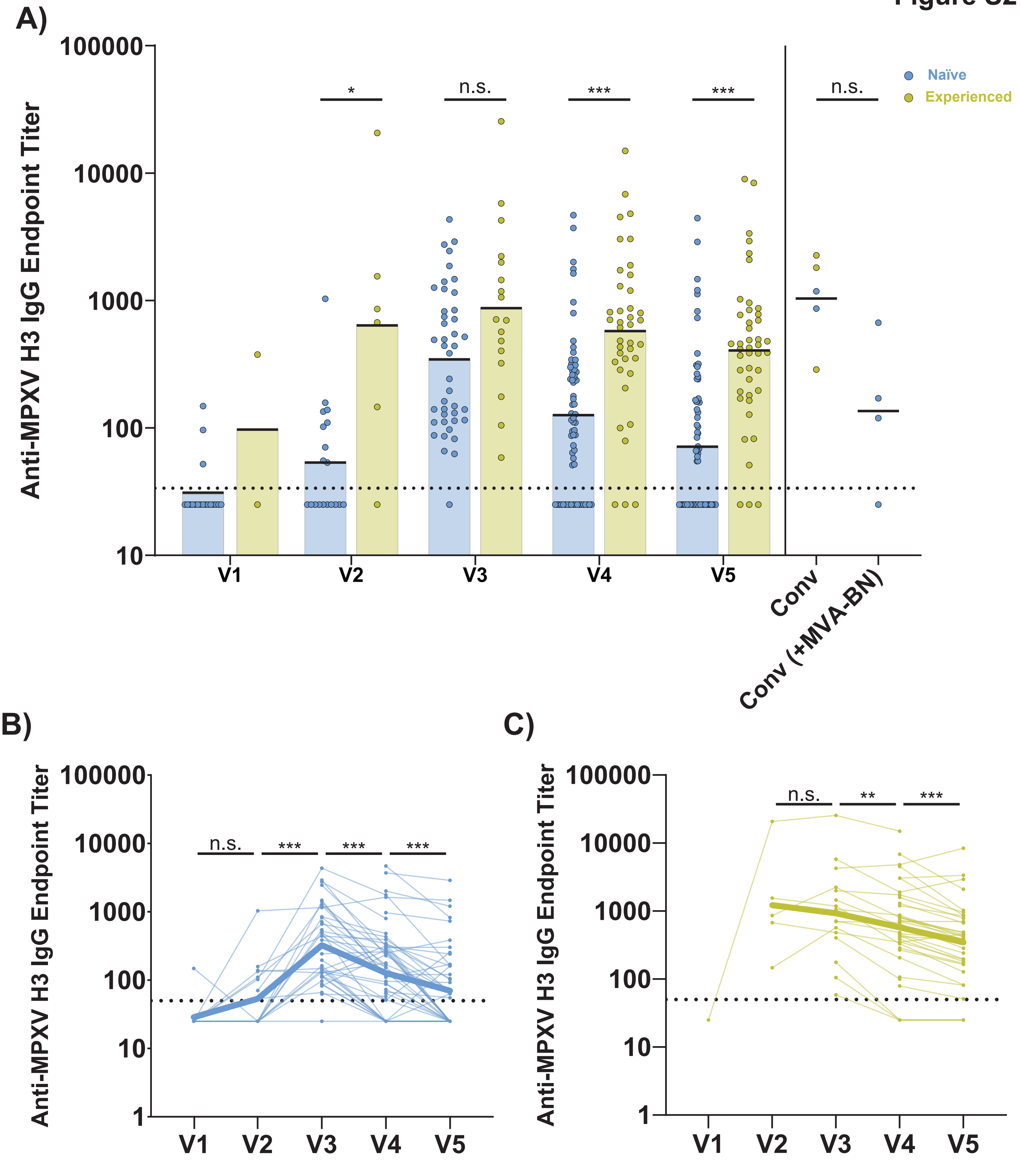

A)
Anti-MPXV H3 IgG Endpoint Titer
100000
10000
1000
100
10
*
n.s.
***
***
n.s.
Naïve
Experienced
V1
V2
V3
V4
V5
Conv
Conv (+MVA-BN)
B)
Anti-MPXV H3 IgG Endpoint Titer
100000
10000
1000
100
10
1
n.s.
***
***
***
V1
V2
V3
V4
V5
C)
Anti-MPXV H3 IgG Endpoint Titer
100000
10000
1000
100
10
1
n.s.
**
***
V1
V2
V3
V4
V5

Figure S2: Anti-MPXV H3 binding titers follow the general trends of MPXV neutralizing titers. A) Serum anti-MPXV H3 IgG titers were measured by ELISA across all participants and study visits. Colored/black bars on each group indicate the geometric mean titer. Horizontal, dashed black line indicates the limit of detection (endpoint titer of 50) for the assay. Any values below the LoD are denoted as an endpoint titer of 25. B and C) Paired anti-MPXV H3 IgG endpoint titers by visit for naïve (B) or experienced (C) participants. Bolded line in panels B and C indicates the mean titer at each time point. Statistical testing conducted by Kruskal-Wallis test with Dunn’s method for multiple comparisons for panel A, and by Wilcoxon matched-pairs signed rank test for panels B and C.

Figure S3

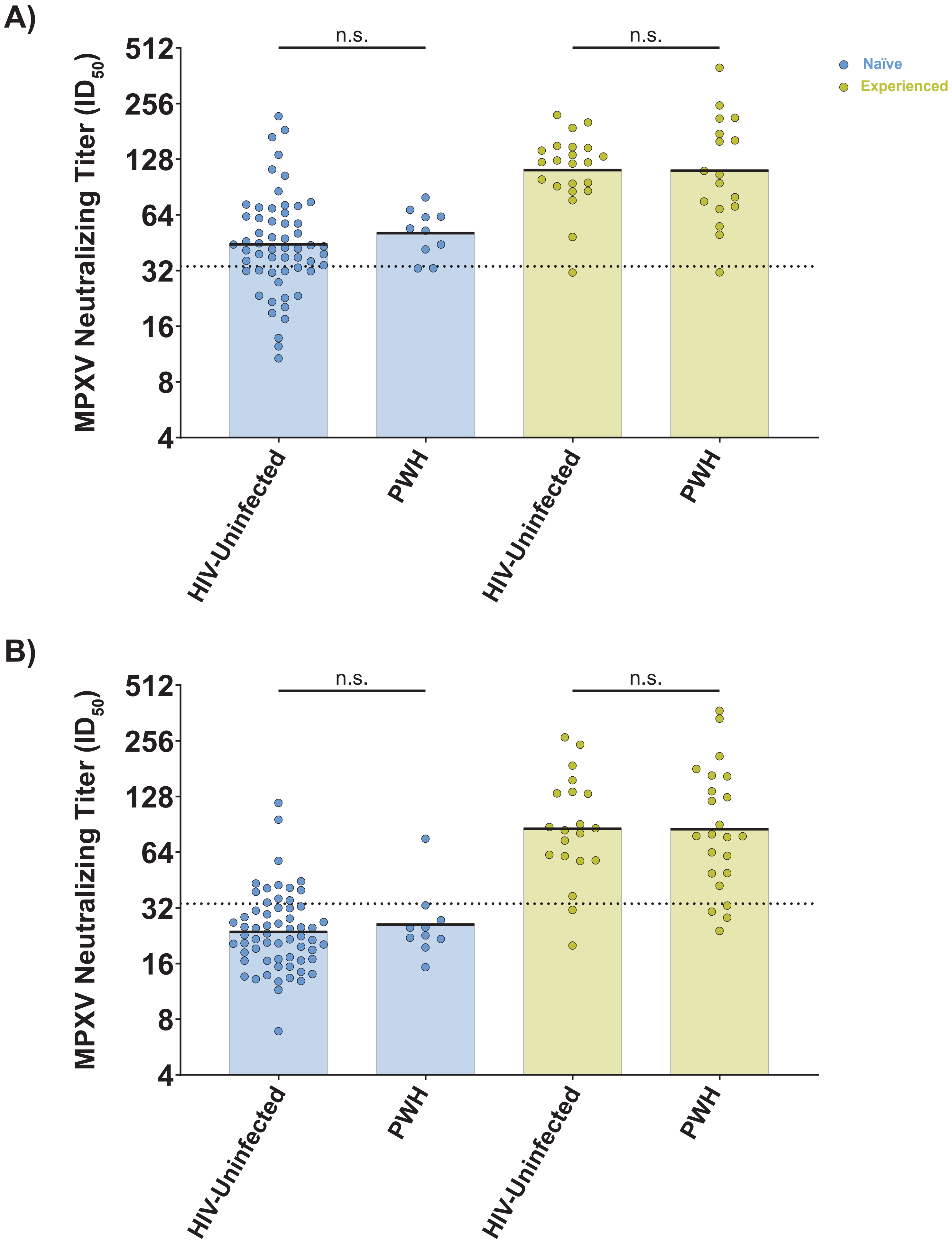
A)
B)
n.s.
n.s.
MPXV Neutralizing Titer (ID50)
512
256
128
64
32
16
8
4
HIV-Uninfected
PWH
HIV-Uninfected
PWH
Naïve
Experienced

Figure S3: Neutralizing titers elicited by MVA-BN are statistically similar between PWH and HIV-uninfected individuals. A) Aggregate MPXV neutralizing titer data for V4 stratified first by prior smallpox vaccination then by HIV status. Colored/black bars on each group indicate the geometric mean titer. Serum neutralizing titers were measured by a fluorescence-based microneutralization assay using authentic clade IIb MPXV. Horizontal, black dashed line in each panel indicates the positivity threshold for neutralization (Fig. 2). B) Same as A, but for V5. Statistical testing conducted by Kruskal-Wallis test with Dunn's method for multiple comparisons.

Figure S4

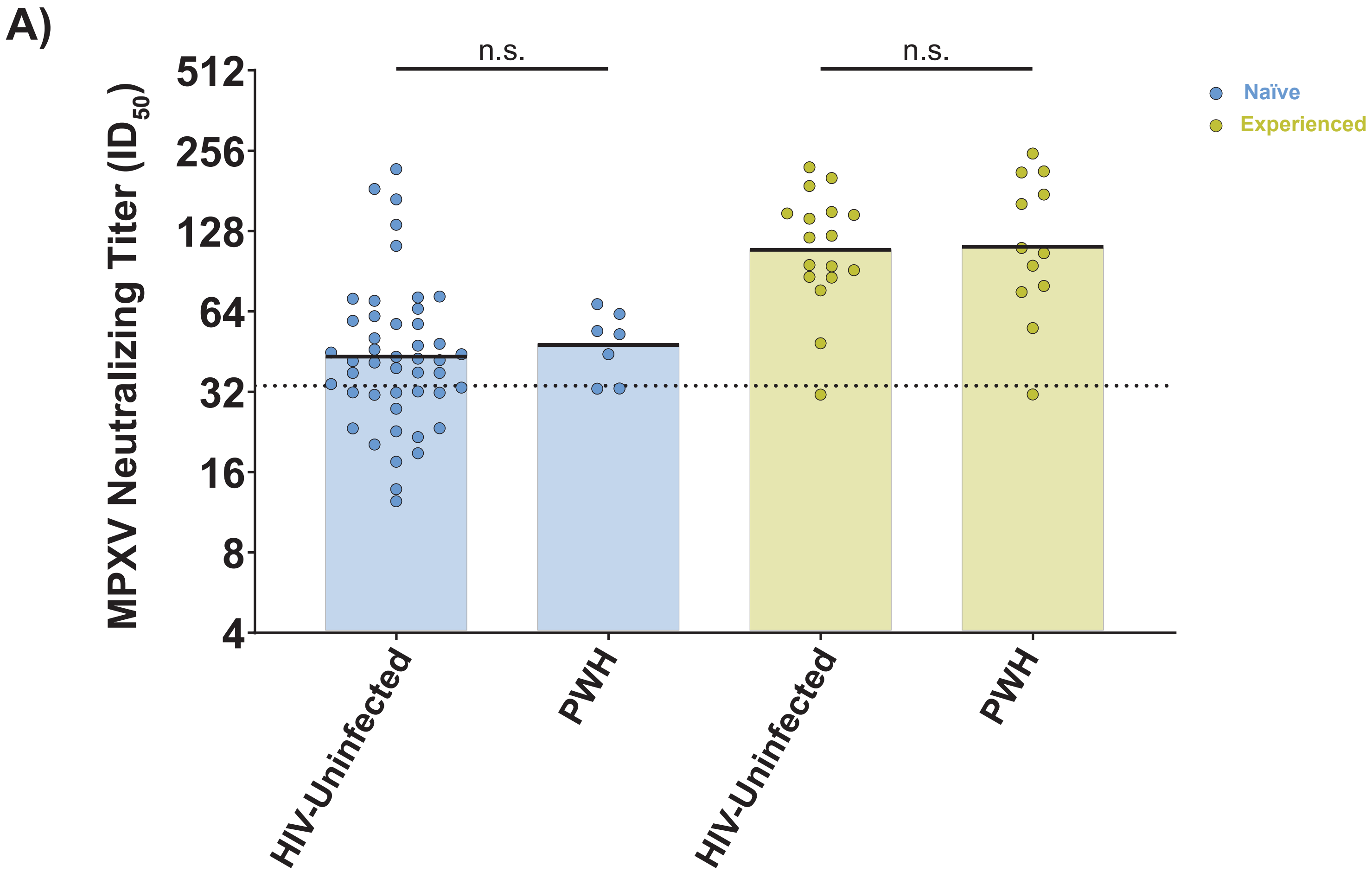

A)
n.s.
n.s.
Naïve
Experienced
MPXV Neutralizing Titer (ID_{50})
512
256
128
64
32
16
8
4
HIV-Uninfected
PWH
HIV-Uninfected
PWH

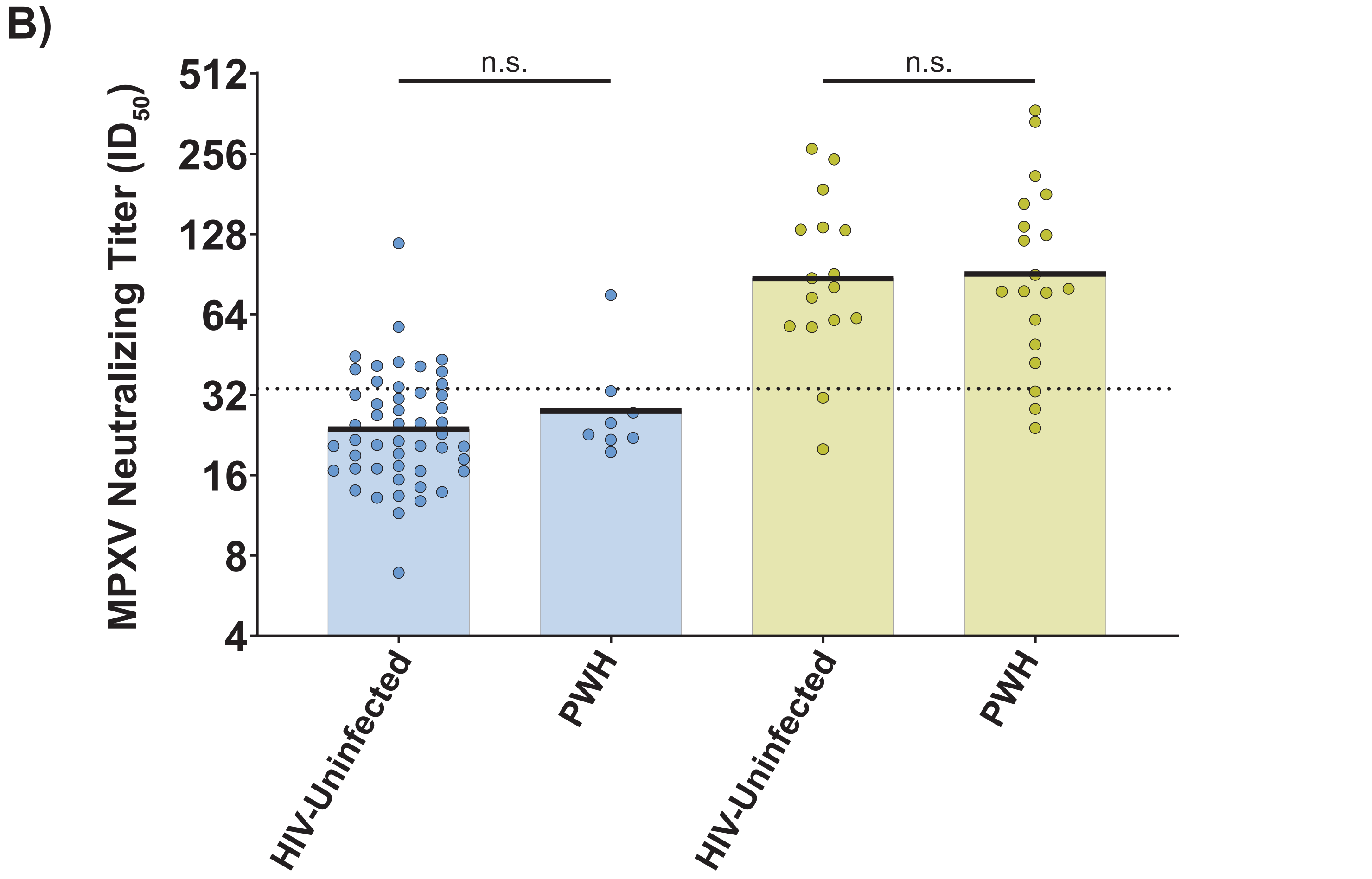

B)
n.s.
n.s.
MPXV Neutralizing Titer (ID_{50})
512
256
128
64
32
16
8
4
HIV-Uninfected
PWH
HIV-Uninfected
PWH

Figure S4: Neutralizing titers elicited by intradermal MVA-BN are statistically similar between PWH and HIV-uninfected individuals. A) Aggregate MPXV neutralizing titer data of participants that received at least one dose of MVA-BN intradermally. Titers for V4 are stratified first by prior smallpox vaccination then by HIV status. Colored/black bars on each group indicate the geometric mean titer. Serum neutralizing titers were measured by a fluorescence-based microneutralization assay using authentic clade IIb MPXV. Horizontal, black dashed line in each panel indicates the positivity threshold for neutralization (Fig. 2). B) Same as A, but for V5. Statistical testing conducted by Kruskal-Wallis test with Dunn's method for multiple comparisons.

Figure S5

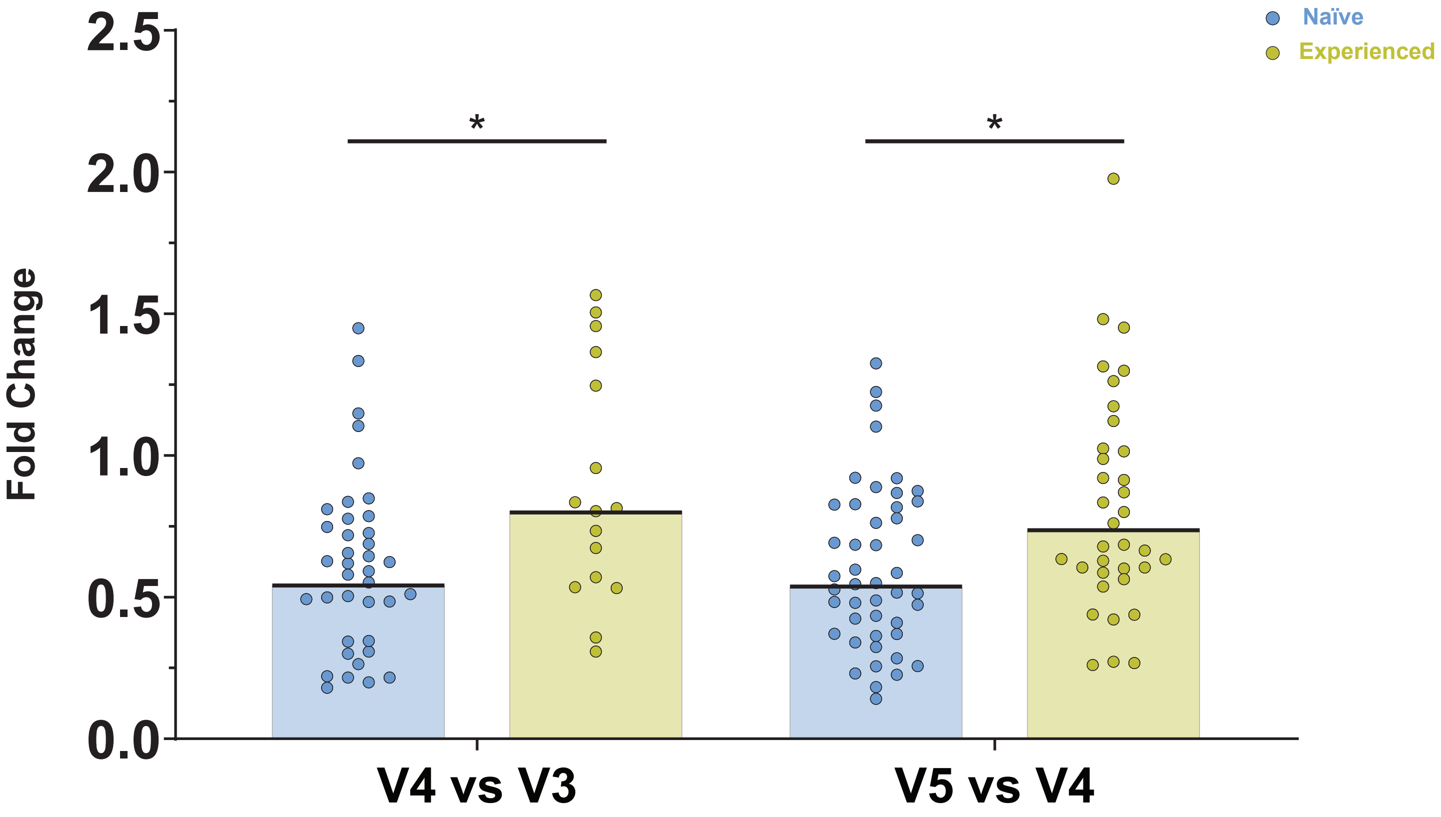

Naïve
Experienced
2.5
2.0
1.5
1.0
0.5
0.0
Fold Change
*
*
V4 vs V3
V5 vs V4

Figure S5: MPXV neutralizing titers wane significantly faster in naïve MVA-BN vaccinees. Comparison of the paired fold changes in neutralizing titers from Fig. 3A and B. Colored/black bar for each group indicates the geometric mean fold change. Statistical testing conducted by Kruskal-Wallis test with Dunn's method for multiple comparisons.

Figure S6

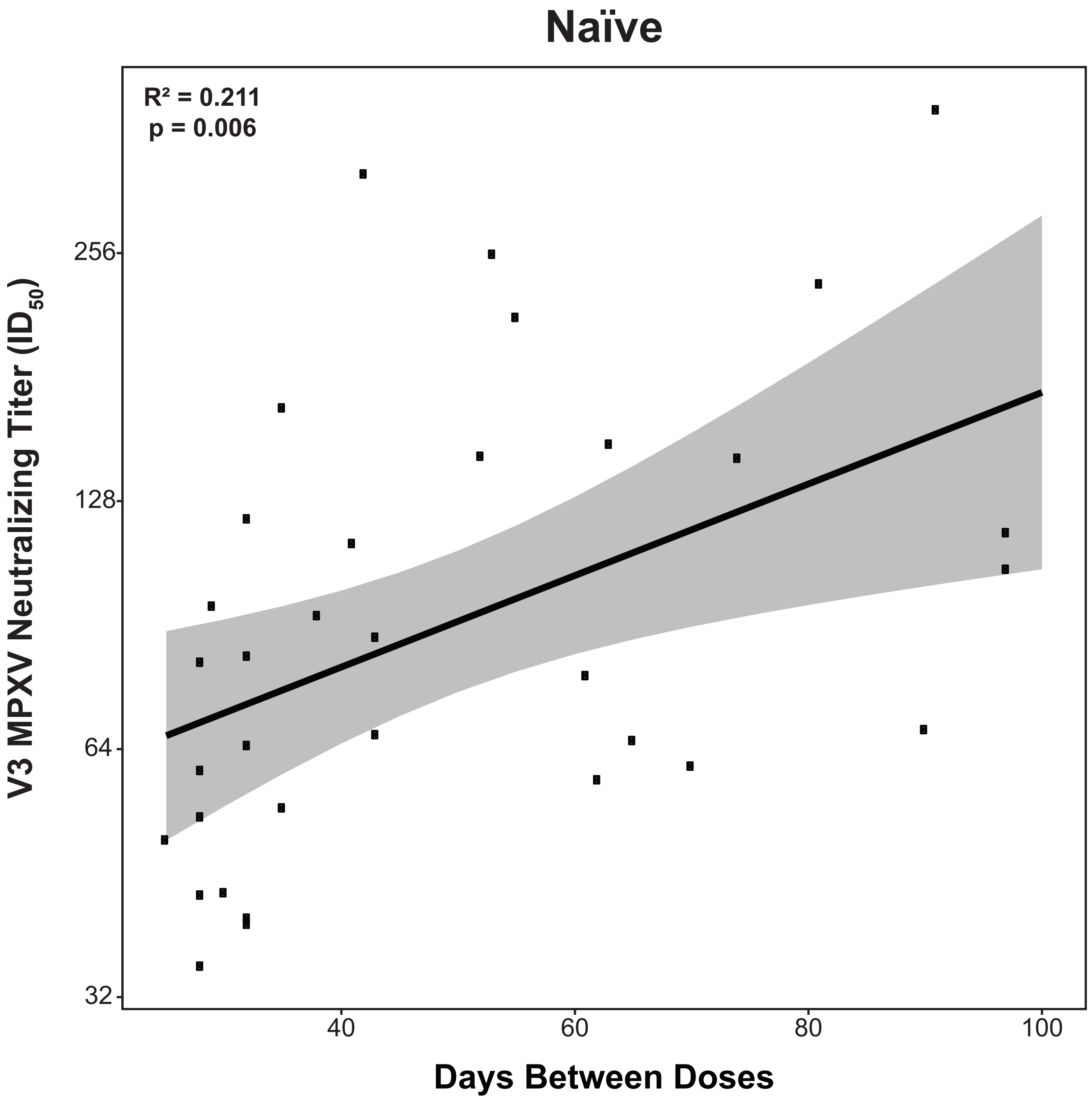

Naïve
R² = 0.211
p = 0.006
V3 MPXV Neutralizing Titer (ID50)
256
128
64
32
40
60
80
100
Days Between Doses

Figure S6: Longer dosing intervals significantly correlate with peak MPXV neutralizing titers in naïve MVA-BN vaccinees. Linear regression of V3 MPXV neutralizing titers versus dosing interval in VACV-naïve participants.

Figure S7

A)

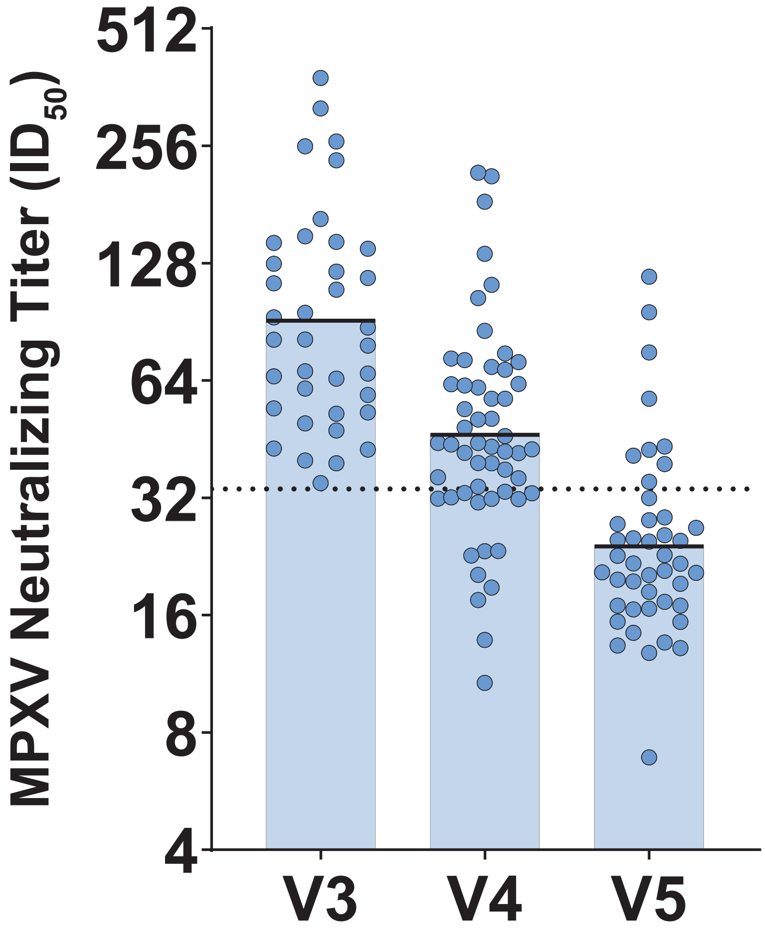

	Naïve V3	Naïve V4	Naïve V5
N	37	54	44
GMT (95% CI)	91.1 (73.9-112.3)	46.4 (39.2-54.9)	24.0 (20.4-28.3)
Median DPV2 (IQR)	19 (15-25)	82 (72-97)	294 (275-304)
% Neg	0	29.6	81.8

B)

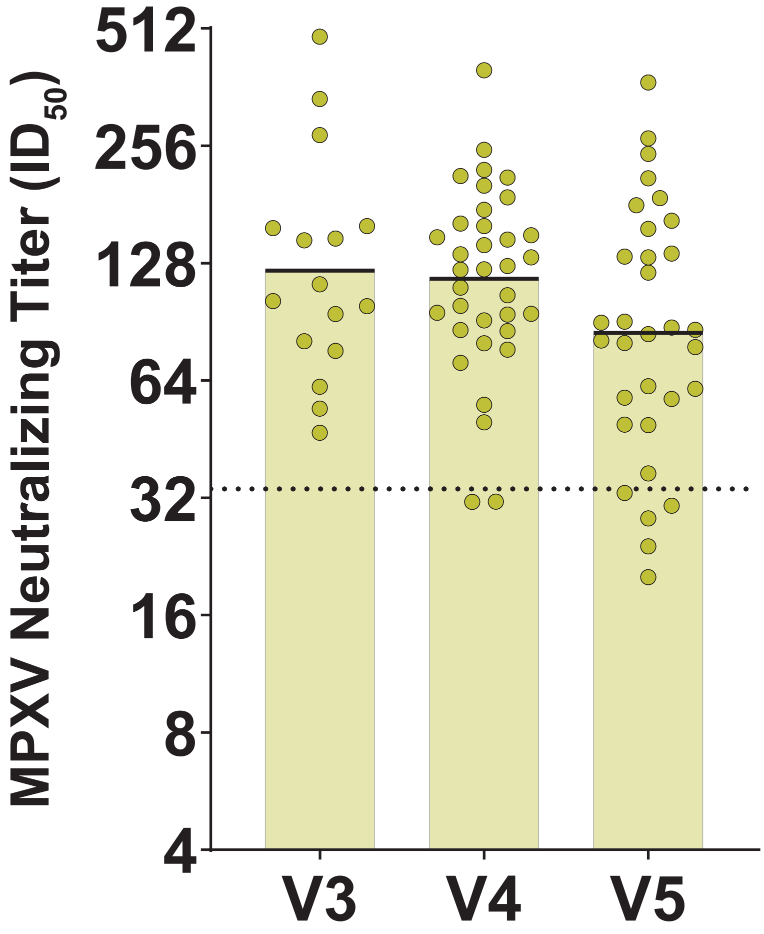

	Exp V3	Exp V4	Exp V5
N	16	35	32
GMT (95% CI)	122.4 (86.5-173.3)	116.7 (96.7-140.8)	84.8 (65.0-110.7)
Median DPV2 (IQR)	21 (18-29)	81 (74-92)	286 (267-313)
% Neg	0	5.7	15.6

● Naïve ● Experienced

Figure S7: Subsets for multiplexed immunoassay analysis have similar neutralization to overall study groups. A) Aggregate MPXV neutralizing titer data for naïve group subset used in Figures 5 and 6. Serum neutralizing titers were measured by a fluorescence-based microneutralization assay using authentic clade IIb MPXV. Colored/black bars on each group indicate the geometric mean titer. Horizontal, black dashed line indicates the positivity threshold for neutralization (Fig. 2). B) Same as A, but for experienced participants. Exp, experienced vaccinees (prior history of smallpox vaccination).

Figure S8

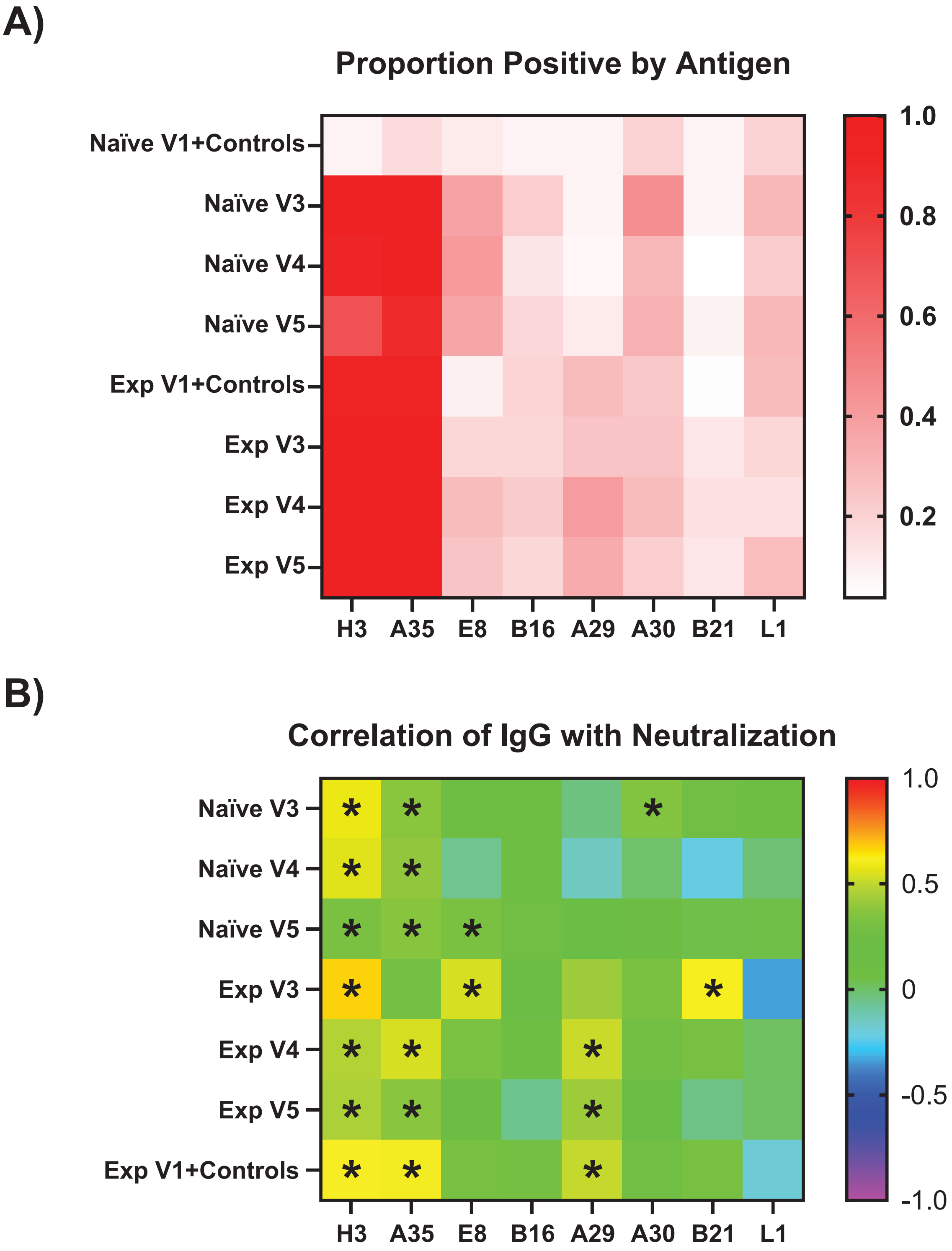
A)
Proportion Positive by Antigen
Naïve V1+Controls
Naïve V3
Naïve V4
Naïve V5
Exp V1+Controls
Exp V3
Exp V4
Exp V5
H3
A35
E8
B16
A29
A30
B21
L1
1.0
0.8
0.6
0.4
0.2
B)
Correlation of IgG with Neutralization
Naïve V3
Naïve V4
Naïve V5
Exp V3
Exp V4
Exp V5
Exp V1+Controls
H3
A35
E8
B16
A29
A30
B21
L1
1.0
0.5
0
-0.5
-1.0

Figure S8: MPXV H3 and A35 are immunogenic in all participants and significantly correlate with MPXV neutralization. Multiplexed immunoassay characterization of samples summarized in Supplementary Figure S7. A) Proportion of participants that are IgG-positive for the indicated MPXV protein at a given time point and prior smallpox vaccination status. B) Spearman correlation for each MPXV protein against neutralizing titers by time point and prior smallpox vaccination status. Significant correlations, i.e., $p < 0.05$, are denoted with an asterisk. Exp, experienced vaccinees (prior history of smallpox vaccination).
